# Machine learning solutions for integrating partially overlapping genetic datasets and modelling host–endophyte effects in ryegrass (*Lolium*) dry matter yield estimation

**DOI:** 10.3389/fpls.2025.1543956

**Published:** 2025-05-06

**Authors:** Jiashuai Zhu, M. Michelle Malmberg, Maiko Shinozuka, Renata M. Retegan, Noel O. Cogan, Joe L. Jacobs, Khageswor Giri, Kevin F. Smith

**Affiliations:** ^1^ Faculty of Science, The University of Melbourne, Parkville, VIC, Australia; ^2^ Agriculture Victoria, AgriBio Centre, Bundoora, VIC, Australia; ^3^ School of Applied Systems Biology, La Trobe University, Bundoora, VIC, Australia; ^4^ Agriculture Victoria, Ellinbank, VIC, Australia; ^5^ Agriculture Victoria, Hamilton, VIC, Australia

**Keywords:** dataset calibration, multidimensional scaling alignment, Procrustes transformation, imputation for structural missingness, population genomics, endophyte effects, plant breeding

## Abstract

Plant genetic evaluation often faces challenges due to complex genetic structures. Ryegrass (*Lolium*), a valuable species for pasture-based agriculture, exhibits heterogeneous genetic diversities among base breeding populations. Partially overlapping datasets from incompatible studies and commercial restrictions further impede outcome integration across studies, complicating the evaluation of key agricultural traits such as dry matter yield (DMY). To address these challenges: (1) we implemented a population genotyping approach to capture the genetic diversity in ryegrass base cultivars; (2) we introduced a machine learning-based strategy to integrate genetic distance matrices (GDMs) from incompatible genotyping approaches, including alignments using multidimensional scaling (MDS) and Procrustes transformation, as well as a novel evaluation strategy (BESMI) for the imputation of structural missing data. Endophytes complicate genetic evaluation by introducing additional variation in phenotypic expression. (3) We modelled the impacts of nine commercial endophytes on ryegrass DMY, enabling a more balanced estimation of untested cultivar–endophyte combinations. (4) Phylogenetic analysis provided a pseudo-pedigree relationship of the 113 ryegrass populations and revealed its associations with DMY variations. Overall, this research offers practical insights for integrating partially overlapping GDMs with structural missing data patterns and facilitates the identification of high-performing ryegrass clades. The methodological advancements—including population sequencing, MDS alignment via Procrustes transformation, and BESMI—extend beyond ryegrass applications.

## 1 Introduction

Plant genetics faces challenges in evaluating agricultural traits, particularly due to the genetic diversity inherent in many plant species, especially outcrossing and polyploid plants (Du et al., 2024; Fu, 2015; Guo et al., 2018) (Barre et al., 2022; Pasquali et al., 2022; Pembleton et al., 2016). Against this backdrop, ryegrass (*Lolium*) is the predominant forage grass genus in many temperate regions worldwide, valued for its high productivity and nutritional value (Andersen et al., 2006; Leddin et al., 2022; Lee et al., 2019; Merino et al., 2019). Its genetic variation is complex due to its outcrossing nature and the admixture of diverse genetic backgrounds from European and North African germplasm in breeding cultivars adapted to Australia and New Zealand (Blackmore et al., 2016; Barre et al., 2022; Guan et al., 2017; Pembleton et al., 2018).

The expression of genetic variation for dry matter yield (DMY), a key performance trait in forage crops, varies considerably across seasons, environments, and management practices. This variability necessitates regional cultivar evaluation systems to provide rigorous and objective assessments of relative performance (Chapman et al., 2017, 2015; Gilliland et al., 2021; Hearn et al., 2021; Leddin et al., 2018; McEvoy et al., 2011).

Advanced statistical methods, such as Linear Mixed Models (LMMs) (Robinson, 1991), have been implemented in analysing multiharvest, multisite (MHMS) field trials to provide accurate prediction for DMY (Giri et al., 2019; Zhu et al., 2023). However, phenotypic data alone cannot account for the complex genetic variability in DMY, especially given the diversity of ryegrasses and the influence of management and environmental factors on trait expression. Addressing these challenges requires advancements in valid genotyping pipelines to generate representative genomic data and the application of appropriate machine learning methods to provide genomic insight into DMY estimation.

Advancements in genotyping approaches have benefited the studies on genetic diversity. While whole genome sequencing remains prohibitively expensive, complexity reduction methods such as genotyping by sequencing (GBS) (Elshire et al., 2011) have facilitated the genetic exploration of ryegrass cultivars using single nucleotide polymorphisms (SNPs) (Keep et al., 2020). Similarly, targeted approaches, such as SNP arrays (Paina et al., 2016; Wang et al., 2014), ensure consistent high coverage of targeted regions across samples.

However, the approaches require further development to address the genetic complexities of ryegrass species. Conventional biallelic discrete encoding cannot accurately represent the high heterozygosity resulting from outcrossing or the multiple chromosome copies in polyploid species (Guo et al., 2018). Addressing these complexities requires the validation of more representative genotyping assays that effectively capture population-level genetic diversity. A pivotal study by Pembleton et al. (2016) introduced a bulk genotyping method using an amplicon-based assay. Multiple individuals from the cultivar with unique genetic backgrounds were bulk-sequenced as a single population, and their genotyping variation was observed to approach zero with increasing bulk size. This strategy effectively captured genetic diversity while providing a high-throughput and cost-effective genotyping pipeline.

**Figure 1:**
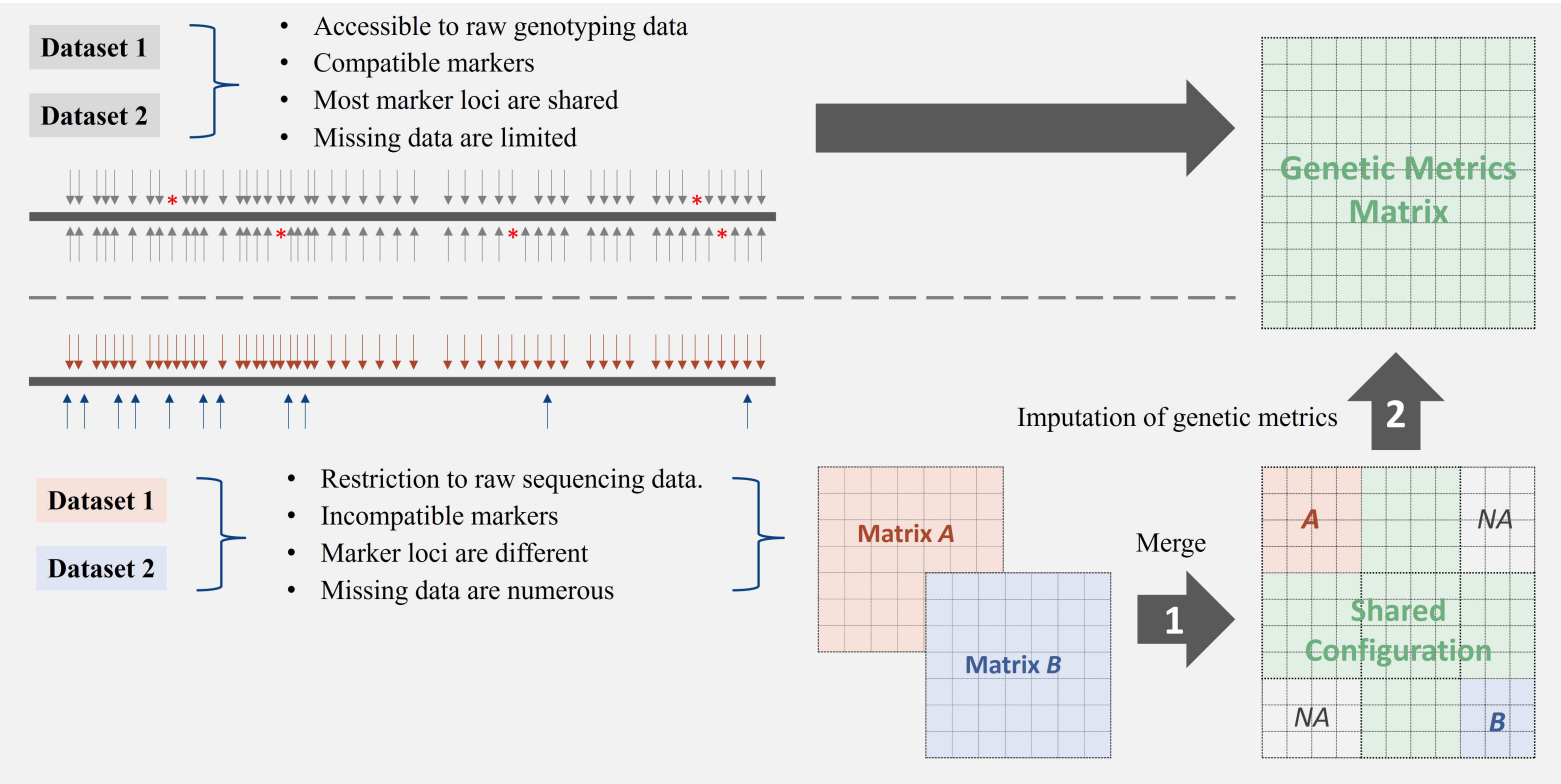
Strategies for integrating genetic datasets with varying compatibility profiles. The top panel illustrates the direct integration of compatible datasets that share common markers and have minimal missing data. The bottom panel depicts a two-step approach (merge + imputation) necessary for integrating incompatible datasets with differing marker loci and substantial missing data. Challenges in matrix integration arise when datasets lack overlapping markers or exhibit structural missingness, requiring specialised imputation methods for genetic distance matrices.

Missing data hinder outcome integration across studies. While current imputation methods address genotype missing data (Browning and Browning, 2016; Browning et al., 2021; Howie et al., 2011; Isik et al., 2017; Jobin et al., 2018; Marchini et al., 2007; Naito and Okada, 2024; Nguyen et al., 2024; Roshyara and Scholz, 2015; Zhao et al., 2022, 2008), they are limited when data originate from incompatible platforms that target different marker types or loci (**Figure 1**), as imputation requires shared marker reference.

Imputation based on derived genetic metrics offers an alternative approach for integrating discrete datasets, particularly genetic distance matrices (GDMs). These symmetric matrices capture genetic relationships between individuals and exhibit patterns reflecting underlying population relatedness (Nei, 1972; Wright, 1949). However, when combining GDMs from independent studies, study-specific samples often result in partially overlapped datasets, where genetic measures for certain samples are completely missing in one study but present in another, leading to structural missingness (**Figure 1**). Imputing the combined GDM presents an issue, as GDMs do not adhere to standard arithmetic rules, making a direct calculation of missing distances from existing pairwise distances challenging.

Machine learning (ML) approaches are increasingly being explored in data integration, yet their application to GDM integration remains limited (Bhattacharjee and Bayzid, 2020; Xia, 2018). Instead, we assumed that each GDM reflects a lower-dimensional snapshot of the true genetic relationship, where relative genetic distances are correctly captured within the limitations of each study. However, inconsistencies in genetic patterns arise across datasets (snapshots) due to variations in genotyping or analytical pipelines. To address these inconsistencies, matrix factorisation and transformation techniques are necessary to align the genetic snapshots across studies. Multidimensional Scaling (MDS) is well-suited for this task, as it preserves distance relationships while reducing dimensionality (Borg and Groenen, 2005; Kruskal and Wish, 1978; Torgerson, 1952) and has demonstrated robustness in applications such as microbiome clustering (Chen et al., 2025) and single-cell multiomics data integration (Chen et al., 2023). Procrustes analysis can align datasets with differing distance scales and has been successfully applied in studies with multidimensional data (Andreella et al., 2023; Gower, 1975; Peres-Neto and Jackson, 2001; Wang and Mahadevan, 2008). Combining these two techniques could provide a promising solution for merging incompatible GDMs. Once merged, the GDM—now an extended genetic snapshot with structural missing values—can be predicted using ML models designed for regression problems, as genetic distances are continuous numeric variables.

In addition, endophytes complicate the estimation of host plant performance. Symbiotic fungi of the genus *Epichloë* are known to alter the phenotypic expression of agronomic traits in ryegrass hosts, enhancing resilience and stabilising field performance, including yield and stress tolerance (Devi et al., 2023; Esqueda et al., 2017). When a ryegrass cultivar is infected with endophyte strains of contrasting genotypes and chemotypes, these grass–endophyte combinations often exhibit distinct agronomic performance (Chapman et al., 2017, 2015; Chen et al., 2020; Devi et al., 2023; Giri et al., 2019; Zhu et al., 2023). Therefore, disentangling the effects of endophytes on host plant performance is crucial for accurate estimation.

Considering the above, this study aimed to validate a genotyping pipeline for representing genetic diversity in ryegrass species, explore ML approaches for integrating partially overlapping GDMs while addressing structural missingness and investigate the impact of endophytes on DMY to provide a more balanced evaluation. Our goal was to provide a high-resolution phylogenetic–phenotypic insight for ryegrass DMY variations. These advancements contribute to a more comprehensive understanding of genetic diversity and its association with DMY assessment in ryegrass.

## 2 Materials and methods

### 2.1 Population sequencing and genotyping

#### 2.1.1 Plant materials

Seeds for all experiments described were obtained from the Australian Pastures Genebank (APG), Australian seed companies that own the respective cultivars, or were commercially purchased. Full details of the germplasm used, including their ploidy types, species, and breeders, are provided in **Supplementary File 1**.

#### 2.1.2 Target capture assay design

A target capture assay was designed using SNPs identified from transcriptome-based GBS (GBS-t) of perennial ryegrass (Malmberg et al., 2018). These genic SNPs were mapped to the Kyuss whole genome reference sequence (Frei et al., 2021) via BLAST to determine their genomic positions. Target capture probes, each spanning 120 basepairs (bp), were designed from the SNPs with reliably identified genomic positions in Kyuss. Overlapping probes were removed, yielding a final set of 27,469 probes collectively covering 71,763 SNPs from the original input list.

#### 2.1.3 Method validation

Several batches of DNA extractions were performed to validate the method, including “Kidman plant bulk sequencing”, “Kidman seed bulk sequencing”, “Rohan seed bulk sequencing”, and 96 batches of individual plant sequencing of Kidman.

Samples were frozen in liquid nitrogen, ground using a Geno/Grinder (Spex SamplePrep, Metuchen, New Jersey, USA), and DNA was extracted using the sbeadex Mini Plant DNA Purification Kit (LGC Biosearch Technologies, Hoddesdon, England) following the manufacturer’s instructions. Sample libraries were prepared with the Twist NGS Library Preparation Kits (Twist Bioscience, South San Francisco, California, USA). All samples were sequenced on an Illumina MiSeq using MiSeq Reagent Kit v2 Micro (Illumina, San Diego, California, USA).

Raw sequencing data were quality-controlled (-q 20) and adapter-trimmed using QuadTrim (v2.0.2) (bitbucket.org/arobinson/quadtrim) and Cutadapt (v0.38) (Martin, 2011). Trimmed reads were aligned to a Kyuss reference genome (Frei et al., 2021) using BWA-MEM (v0.7.17) (Li, 2013) and sorted using SAMtools (v1.9) (Danecek et al., 2021). The sorted sequences from multiple flow cells were merged; duplicates were marked using GATK (v4.1.3) (Van der Auwera and O’Connor, 2020). BCFtools (v1.9) (Danecek et al., 2021) was used to call variants from the SNP list successfully transferred from the GBS-t data to the Kyuss whole genome reference. Indels were excluded, and the resulting Variant Call Format (VCF) data were normalised. Only SNPs where both the reference and alternative alleles matched those specified in the SNP list were retained. SNP calls were subsequently filtered to retain loci with read depths (DP) > 5 and a percentage of missing data < 5%, as well as samples with missing data < 20%.

Population genotypes were derived by calculating reference allele frequencies from the allelic depth (AD) field using vcfR (v1.15.0) (Knaus and Grünwald, 2017). Nei’s GDMs (Nei, 1972; Pembleton et al., 2016) were calculated from these allele frequencies using the R StAMPP package (v1.6.3) (Pembleton et al., 2013) and were used to construct an unrooted tree with the R ggtree package (v3.10.0) (Yu et al., 2017). This approach validated the population sequencing method and helped determine the optimal number of individuals required to represent a single genotyping population. Further details on the validation process are available in **Supplementary File 2**.

#### 2.1.4 Population sequencing and genotyping

A minimum of 50 seeds per cultivar were bulk-sequenced using an Illumina NovaSeq 6000 with an S4 flow cell (Illumina, San Diego, California, USA), as described above. The population genotypes of 80 cultivars were derived following the same approach and aggregated into 72 unique genotypes by averaging allele frequencies per locus across the populations with the same host genetic background. This aggregation ensured that each genotype uniquely represented a single base ryegrass population for further analysis.

### 2.2 Integration of partially overlapped genetic distance matrices

#### 2.2.1 Datasets

##### 2.2.1.1 Ryegrass72_2024

A Nei’s GDM denoted as A , was derived from the 72 unique ryegrass genotypes using the R StAMPP package (v1.6.3) (Pembleton et al., 2013).

##### 2.2.1.2 Ryegrass63_2016

Ryegrass63_2016 dataset, denoted as B , is an existing Nei’s GDM representing relationships among 63 ryegrass populations (Pembleton et al., 2016), including perennial ryegrass, Italian ryegrass, hybrid ryegrass, and Festulolium cultivars. This dataset was derived from a GBS pipeline based on 296 SNP loci (from an initial set of 380, which were defined by the Illumina GoldenGate OPA), resequenced using a targeted amplicon approach on bulk seed and leaf samples via the Illumina HiSeq 2000 platform.

#### 2.2.2 Multidimensional scaling alignment via Procrustes transformation

The pairwise genetic distances in each GDM were assumed to provide a lower-dimensional snapshot of the true genetic relationship among the tested populations, where genotyping approaches correctly captured the relative relationship within each study. With this assumption, the two GDMs A and B were projected into Euclidean spaces, as $E_{A}$ and $E_{B}$ respectively, by multidimensional scaling (MDS) (Gower, 1966; Torgerson, 1952) using “cmdscale” (v4.4.0) (R Core Team, 2024). Eigen-decomposition was performed on the centred Gram matrices $-\frac{1}{2}H_{A}A^{2}H_{A}$ and $-\frac{1}{2}H_{B}B^{2}H_{B}$ , where $H_{A}$ and $H_{B}$ are the double-centring matrices of A and B . We obtained


$$X=V_{A,\zeta }\Lambda_{A,\zeta }^{\frac{1}{2}}\;\mathrm{and}$$

$$Y=V_{B,\zeta }\Lambda_{B,\zeta }^{\frac{1}{2}}$$

where ***X*** and ***Y*** denote the MDS configurations of A and B , respectively; $V_{A,\zeta }$ and $V_{B,\zeta }$ are the eigenvectors; $\Lambda_{A,\zeta }$ and $\Lambda_{B,\zeta }$ are the diagonal matrices containing the non-negative eigenvalues; ζ is smallest number of MDS dimensions that retain non-negative eigenvalues across $E_{A}$ and $E_{B}$ .

***Y*** was subsequently subjected to Procrustes transformation, using “procrustes” in an R package “vegan” (v2.6-6.1) (Oksanen et al., 2024), based on the common genotypes ( γ ) with ***X*** to obtain a calibrated MDS configuration (as $Y^{\prime }$ ) by rotating, scaling, and translating:


$$Y^{\prime }=sYR+\tau$$

where, $Y^{\prime }$ is the transformed MDS configuration of ***Y***; s is a scaling factor calculated by the ratio of the Frobenius norms of $\left(X_{\gamma \zeta }-{\bar{X}}_{\zeta }\right)$ over $\left(Y_{\gamma \zeta }-{\bar{Y}}_{\zeta }\right)$ **;** $X_{\text{γζ}}$ and $Y_{\gamma \zeta }$ are the shared configurations of ***X*** and ***Y***, ${\bar{X}}_{\zeta }$ and ${\bar{Y}}_{\zeta }$ are the respective centroids, and γ=22, ζ=33 **;** τ is a translation vector calculated by ${\bar{X}}_{\zeta }-s{\bar{Y}}_{\zeta }R$ ; R is an orthogonal rotation matrix given by $UV^{T}$ **;** U and V were the respective left and right singular vectors obtained from the singular value decomposition (SVD) of the cross-covariance matrix ${\left(X_{\gamma \zeta }-{\bar{X}}_{\zeta }\right)}^{T}\left(Y_{\gamma \zeta }-{\bar{Y}}_{\zeta }\right)$ .

After obtaining $Y^{\prime }$ , it was mapped back to Nei’s genetic space as $B^{\prime }$ by computing pairwise Euclidean norms. Finally, the two matrices A and $B^{\prime }$ were merged by averaging the distance values of shared populations and keeping the distance values of unique populations, to acquire an extended matrix denoted as M .

#### 2.2.3 Imputation of structural missingness in the partially overlapped genetic datasets

The matrix M contains 32.1% missing data (NAs), which were imputed as multiple propagated regression problems in this study. To evaluate imputation performance for structured missing data, we introduced a strategy called Bootstrap Evaluation for Structural Missingness Imputation (BESMI). This method introduces structural missingness by randomly assigning NAs to entire rows and columns. Predictive models leverage existing distance patterns to infer missing values while maintaining the integrity of genetic relationships. The replaced values served as the validation set.

Ten distinct training-validating datasets, $M_{m}$ , were generated through five-fold bootstrap sampling. For each dataset $M_{m}$ , 14 populations were randomly selected from ***A*** and 13 populations from ***B***’ (i.e., 14≈15×72 and 13≈15×63 ). Missingness was simulated by replacing the observed values with NAs in the corresponding rows and columns for the selected populations.

The imputation proceeded through multiple chained predictions iteratively (Van Buuren, 2018). In each iteration, the tail-imputed matrix at iteration k for dataset $M_{m}$ . was given as $M_{\left(m,k\right)}^{n}$ , where n is the upper bound at the iteration k , as:


$$M_{\left(m,k\right)}=\{\begin{array}{c} M_{\left(m,k\right)}^{n}\;\;\;\;\;\;,\;\;k=1\\ \frac{M_{\left(m,k\right)}^{n}+M_{\left(m,k-1\right)}^{n}}{2},\;\;k>1 \end{array}$$

As ***M*** is pairwise and symmetric, the imputation was performed by a column-wise prediction for the populations with NAs. Population j was predicted from the observed or propagated predictions of the other populations. Let $z_{\left(i,j\right)}$ denote the distance between the population j and the population i , and let ${\hat{z}}_{\left(i,j\right)}$ denote its predicted distance. If $z_{\left(i,j\right)}$ is NA, the imputed value ${\hat{z}}_{\left(i,j\right)}^{k}$ at iteration k was computed as:


$${\hat{z}}_{\left(i,j\right)}^{k}=f\left(\left\{z_{\left(i,1\right)},z_{\left(i,2\right)},\ldots ,z_{\left(i,p\right)}\right\}\cup^{\;}\left\{{\hat{z}}_{\left(i,1\right)}^{k},{\hat{z}}_{\left(i,2\right)}^{k},\ldots ,{\hat{z}}_{\left(i,q\right)}^{k}\right\}\right)$$

where f is the prediction function; p and q are the indices of observed and predicted populations, respectively; p+q=112 in a fully imputed matrix.

The predictions f , including Regression Tree (RT), Random Forest (RF), Lasso Regression (Lasso), K-Nearest Neighbours (KNN), Predictive Mean Matching (PMM), Weighted Predictive Mean Matching (WPMM), Random Sampling (SAMPLE), and Mean (Baseline), were achieved using “mice” (v3.16.0) (Van Buuren and Groothuis-Oudshoorn, 2011) and “DMwR2” (Torgo, 2011). The imputation accuracy was assessed by the Coefficient of Determination ( ${\text{R}}^{2}$ ) by comparing the imputed values ( ${\hat{z}}_{\left(i,j\right)}$ ) with the actual values ( $z_{\left(i,j\right)}$ ) initially replaced with NAs in each dataset $M_{m}$ . Details of the imputation process and the prediction methods f can be found in **Supplementary File 3**.

Based on ${\text{R}}^{2}$ , Lasso regression was selected to impute the missing Nei’s genetic distances in M . The imputed values were averaged with their corresponding symmetric counterparts to maintain matrix symmetry. The resulting imputed matrix $M^{*}$ provided a complete set of pairwise Nei’s genetic distances for the 113 populations.

### 2.3 Estimation of DMY for potential host–endophyte combinations

#### 2.3.1 Field trial data

Phenotypic characteristics (DMY in kg DM/ha per harvest) of the ryegrass cultivars were analysed using an MHMS dataset, which was previously used to study the genotype-by-environment interaction of perennial ryegrass in southeastern Australia (Zhu et al., 2023). The dataset consists of 18 MHMS pasture trials conducted over 14 years (2008–2021) for 126 perennial ryegrasses, Italian ryegrasses, and Festulolium cultivars.

#### 2.3.2 Statistical modelling

The MHMS data were fitted by linear mixed models (LMMs) using ASReml-R (v3.00) (Butler et al., 2009) to reveal the phenotypic characteristics and the impacts of nine commercial endophytes (plus Nil, denoting low or no endophytes) on the DMY of the base ryegrass cultivars explored above. The endophyte effects were modelled as fixed or random effects.

The LMMs were given as:


y=Xβ+Zu+ϵ

where y is a column vector containing the observations; X is the design matrix for fixed effects; β is the column vector of fixed coefficients; Z is the design matrix for random effects; u is the vector of random coefficients; ϵ is the vector of residual errors.

##### 2.3.2.1 The base model (LMM0)

The models without accounting for *endophyte* effects were treated as the base model (LMM0) fitted by the factor analytical (FA) strategy (Zhu et al., 2023) and were given as:

a. The fixed components


$$X_{0}\beta_{0}=\left(\mu ,T,H\left|T,R\right|\left(H,T\right),C|\left(H,T\right)\right){\left(1,\beta_{T},\beta_{H},\beta_{R},\beta_{C}\right)}^{T}$$

wherein, $X_{0}\beta_{0}$ denotes the fixed components; μ,T,H|T,R|(H,T),C|(H,T) denote the corresponding vectors/matrices for *Intercept*, *Trial*, *Harvest within Trial*, first-order polynomial effect of *Row of the Harvest within Trial*, and first-order polynomial effect of *Column of the Harvest within Trial*, respectively; $1,\beta_{T},\beta_{H},\beta_{R},\beta_{C}$ denote the 39,812×1 vector of *Intercept*, the 18×1 vectors of *Trial* main effects, the 18h×1 vectors of *Harvest* nested effects within a trial ( h varies across trials), the 18h×1 vectors of first-order polynomial effect of *Row of the Harvest within Trial*, and the 18h×1 vectors of first-order polynomial effect of *Column of the Harvest within Trial*, respectively.

b. The random components


$$\text{Var}\left(u_{0}\right)=\left(\Gamma \Gamma^{\text{T}}+\Psi \right)⊗I_{ɡ}+\text{diag}\left(\sigma_{k}^{2}{\left(\sum_{H}^{\text{ar}\left(\lambda \right)}⊗I_{ɡ}\right)}_{k}\right)$$

wherein, Γ denotes latent factors that account for the genetic variances of the environments, and $\Gamma^{\text{T}}$ represents its transpose matrix of Γ ; Ψ contains the unique variances that cannot be accounted for by the FA; $I_{ɡ}$ denote the genotypic variance components of ryegrasses and were assumed $\left\{I_{ɡ\left(i,i\right)}\right\}\sim N\left(0,R_{ɡ}\right)$ , $\left\{I_{ɡ\left(i,j\right)}|\left(i\neq j\right)\right\}=0\;$ . i and j are the indexes of the matrix $I_{ɡ}$ ; $\sum_{H}^{ar\left(\lambda \right)}$ denote the variance components of harvests and were assumed to follow an order -λ autoregressive distribution, λ∈{1,2,3} ; ${\text{σ}}_{k}^{2}$ is the independent variance for trial k .

c. The residual components

Let ϵ denote the residual component for each set of LMMs, ϵ~N(0,Var(ϵ)) , its variances-covariance matrix, Var(ϵ) , was given by separate four-way structures:


$$\text{Var}\left(\epsilon \right)=diag\left(\sigma_{k}^{2}{\left(\Sigma_{H}^{ar\left(\lambda_{H}\right)}\right)}_{k}⊗{\left(\Sigma_{R}^{ar\left(\lambda_{R}\right)}\right)}_{k}⊗{\left(\Sigma_{C}^{ar\left(\lambda_{C}\right)}\right)}_{k}\right)$$

where, ${\text{σ}}_{k}^{2}$ denotes the independent variance component of the trial k ; $\Sigma_{H}^{ar\left(\lambda_{H}\right)}$ , $\Sigma_{R}^{ar\left(\lambda_{R}\right)}$ , and $\Sigma_{C}^{ar\left(\lambda_{C}\right)}$ , are the variance–covariance matrices for harvests, rows, and columns, respectively, and are following order $-\lambda_{H}$ , order $-\lambda_{R}$ , and order $-\lambda_{C}$ autoregressive distribution, $\lambda_{H},\lambda_{R},or\;\lambda_{C}\in \left\{1,2,3\right\}$ .

##### 2.3.2.2 Models where endophytes were fitted as random components

The LMMs (LMM1) to account for endophyte effects across and within the ryegrass populations as random effects, shared the same fixed components ( $X_{1}\beta_{1}$ **=** $X_{0}\beta_{0}$ ) and residual components as the base model (LMM0); the variance components including random endophyte effects were given as:


$$\text{Var}\left(u_{1}\right)=\left(\begin{array}{cc} \text{Var}\left(u_{0}\right) & 0\\ 0 & I_{endo} \end{array}\right)$$

Wherein, $I_{endo}$ denotes the variance components of endophytes and was assumed $\left\{I_{endo\left(i,i\right)}\right\}\sim N\left(0,R_{endo}\right)$ , $\left\{I_{endo\left(i,j\right)}|\left(i\neq j\right)\right\}=0$ .

##### 2.3.2.3 Models where endophyte effects were fitted as fixed effects

The models (LMM2) that fit *endophyte* effects as fixed effects, while accounting for the variability caused by *Harvest*, *Row*, and *Column*, retained the same random ( $\text{Var}\left(u_{2}\right)=\text{Var}\left(u_{0}\right)$ ) and residual components as the base model (LMM0); the fixed effects were given as:


$$X_{2}\beta_{2}=\left(X_{0},N\right){\left(\beta_{0},\beta_{N}\right)}^{\text{T}}$$

Wherein, N denotes the matrices for *endophyte*; $\beta_{N}$ denotes the 10×1 vectors of *endophyte* main effects.

The goodness of fit of the LMMs within each set (i.e., LMM0, LMM1, or LMM2) was evaluated using the natural logarithm of Restricted Maximum Likelihood (logREML), while the goodness of fit across sets was assessed using the Akaike Information Criteria (AIC) (Bozdogan, 1987) and mean squared error (MSE).

The predicted DMYs were analysed by ANOVA (R Core Team, 2024) to assess group differences. Endophyte impacts ( E ) and the DMY of ryegrass populations ( P ), along with their standard errors, were derived from the best-identified LMMs. The DMY estimates for potential host–endophyte combinations ( PE ) were calculated as PE=μ+P+E . Their standard errors ( S.E. ) were derived by aggregating the standard errors of endophyte estimates ( $SE_{x}$ ) and population predictions ( $SE_{y}$ ) as: $S.E.=\sqrt{SE_{x}^{2}+SE_{y}^{2}}$ , assuming mutual independence.

### 2.4 Phylogenetic–phenotypic association of DMY

A phylogenetic tree was constructed from the imputed matrix $M^{*}$ to explore phylogenetic relationships among the 113 ryegrass populations; along with the phenotypic estimation (DMY in kg DM/ha per harvest) and the estimated endophyte effects were visualised using “ggtree” (v3.10.0) (Yu et al., 2017) and “ggforce” (Pedersen, 2024). The estimate ( ν ) of the DMY of each group was given by the weighted average of the DMY of the populations in such group, as:


$$\nu =\frac{\sum_{i=1}^{k}\omega_{i}P_{i}}{\sum_{i=1}^{k}\omega_{i}}$$

The standard error ( s.e. ) of the DMY in each group was given by aggregating the variances of population predictions and the weighted variances among the populations in such group, as:


$$s.e.=\sqrt{\frac{1}{k^{2}}\sum_{i=1}^{k}se_{i}^{2}+\frac{\sum_{i=1}^{k}\omega_{i}{\left(P_{i}-\nu \right)}^{2}}{\sum_{i=1}^{k}\omega_{i}}}$$

where k is the number of populations in such group; $P_{i}$ is the DMY of the population i ; $se_{i}$ is standard error of $P_{i}$ ; ωi=1sei2 , is the inverse of the variance of $P_{i}$ .

The populations whose predictions could not be derived from the LMMs were estimated by the groups they belong to; their standard errors were given by the standard error of the group.

## 3 Results

### 3.1 Validation of population genotyping approaches

The reliability of the population genotyping assay was validated, as shown by the unrooted tree in **Supplementary File 2**. The branches of “Kidman plant bulk sequencing” and “Kidman seed bulk sequencing” were tightly clustered, validating that the sequencing material does not bias the genotyping results. The branches of Kidman individual sequencing (branches 1–10) clustered with the two Kidman bulk sequencing branches (i.e., plant and seed), demonstrating the capability to consistently genotype ryegrasses with the same genetic background when applying different sequencing strategies (i.e., bulk vs. individual); “Rohan seed bulk sequencing” branch was distinctly separated from all the Kidman branches, validating the effectiveness of differentiating ryegrasses with different genetic backgrounds. The genetic distances of Kidman branches were observed to decrease to less than 0.01 when the samples pooled 20 or more individuals.

### 3.2 Population sequencing and genotyping

A total of 80 ryegrass cultivars were successfully genotyped using bulk seed sequencing, with an average coverage depth of 350.81 × across the target regions. After variant calling and filtration, 85,903 valid SNPs were identified, and all samples passed the filtering thresholds.

**Figure 2:**
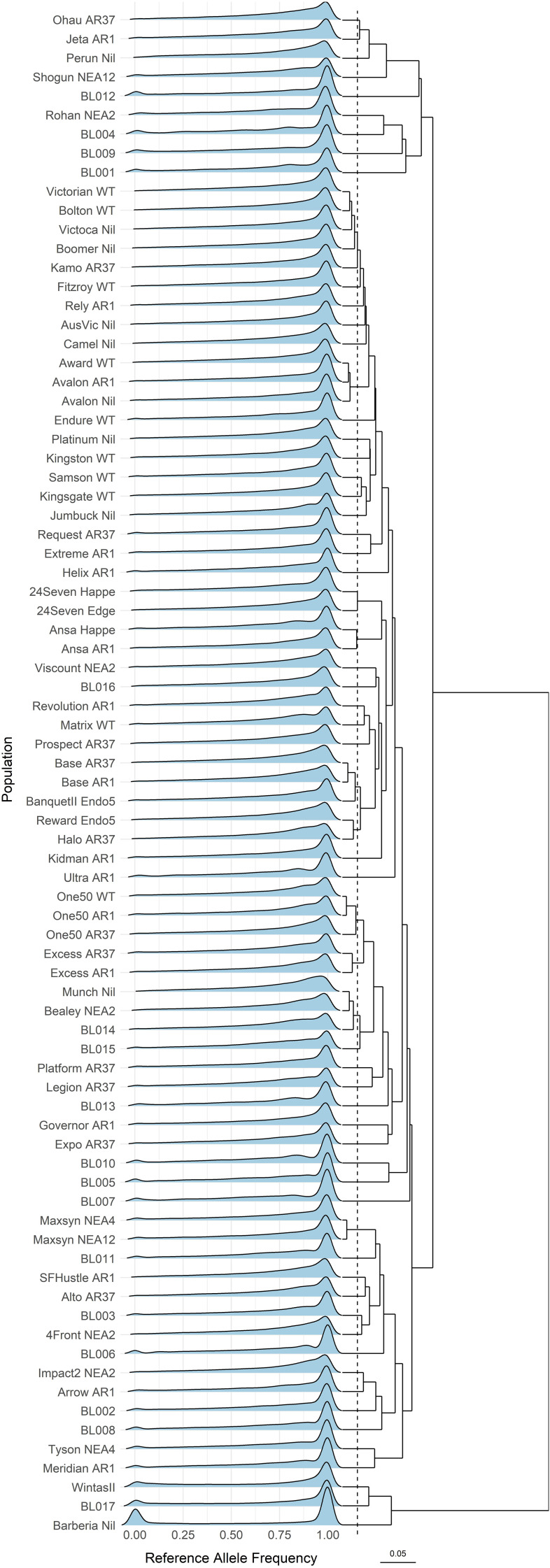
Density ridges of the reference allele frequency distribution across the 80 ryegrass cultivars. The dendrogram represents hierarchical clustering based on Nei’s genetic distances. The dashed line (at Nei’s genetic distance of 0.0182) suggests a potential threshold for populations that share the same genetic background. The scale bar corresponds to 0.05 Nei’s genetic distances.

The genotypes were represented as reference allele frequencies (Zhu et al., 2024), with their distribution (**Figure 2**) revealing cultivars with distinct genetic backgrounds. Cultivars with identical genetic backgrounds but associated with different endophytes clustered closely. For example, 24Seven, Ansa, Avalon, Base, Excess, Maxsyn, and One50 exhibit genetic distances of less than 0.0182 within their corresponding genetic identity. The 80 cultivars were consolidated into 72 base cultivars with unique genetic backgrounds.

### 3.3 Multidimensional scaling alignment via Procrustes transformation

**Figure 3:**
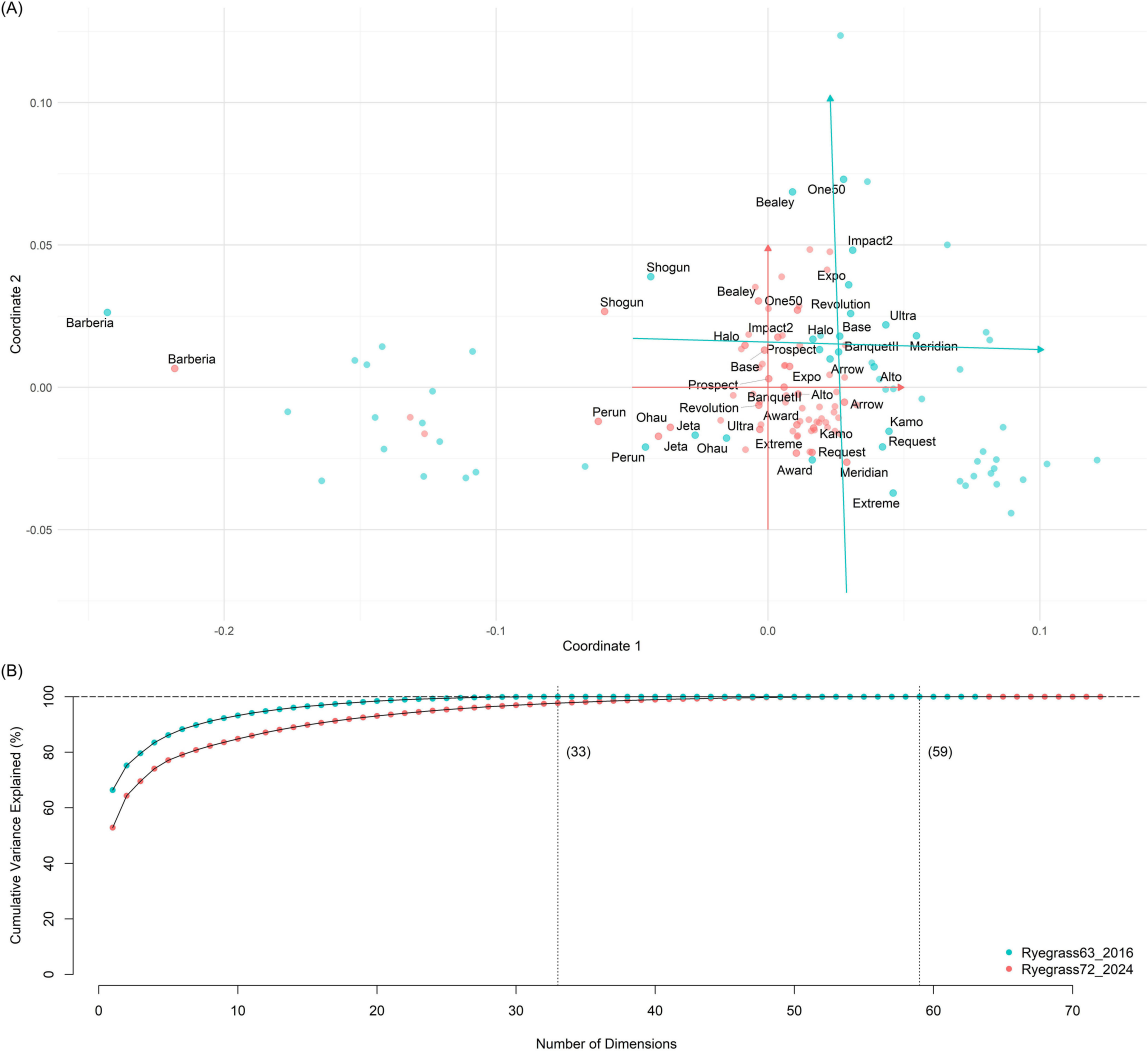
Result visualisation of multidimensional scaling (MDS) alignment via Procrustes transformation. **(A)** Projections of Nei’s genetic distances among 72 ryegrass populations in ***A*** (red) and 63 ryegrass populations in ***B*** (turquoise) on the first two principal coordinates (2PCs) of the MDS configurations of ***X***. The red arrows represent the first 2PCs of ***X***, while the turquoise arrows represent the first 2PCs of ***Y*** before the Procrustes transformation. The lengths of the coordinates correspond to 0.1 genetic units. Only populations present in both datasets are labelled. **(B)** Cumulative variances are explained by a series of MDS configurations for ***X*** (red) and ***Y*** (turquoise). The number of nonnegative eigenvalues contributing to the MDS spaces for ***X*** is 59 and 33 for ***Y***.

The GDM ***A*** and ***B*** were projected into MDS space ***X*** and ***Y***, respectively. A 33-dimensional solution was selected after examining the cumulative explained variances, accounting for 97.7% of the variance in ***A*** and 100% in ***B***. The configurations on the first two principal coordinates of the MDS spaces were illustrated in a scatter plot (**Figure 3**), where the first two principal coordinates accounted for 52.8% and 11.5% of the variance in ***A***, and 66.4% and 8.9% in ***B***, showing the direction and magnitude of the transformation from the MDS configurations of ***Y*** to ***X***.

The bias between ***A*** and ***B*** followed a distribution with a mean of − 0.021, which is 34.4% biased relative to the mean of ***A*** (0.061), while the bias between ***A*** and ***B*** ${\;}^{\prime }$ followed a distribution with a mean of − 0.0003. The sum of squared differences (SSD) was reduced by 73.2%, decreasing from 0.284 before calibration to 0.076 after calibration.

### 3.4 Imputation of structural missingness in the partially overlapping genetic datasets

**Figure 4:**
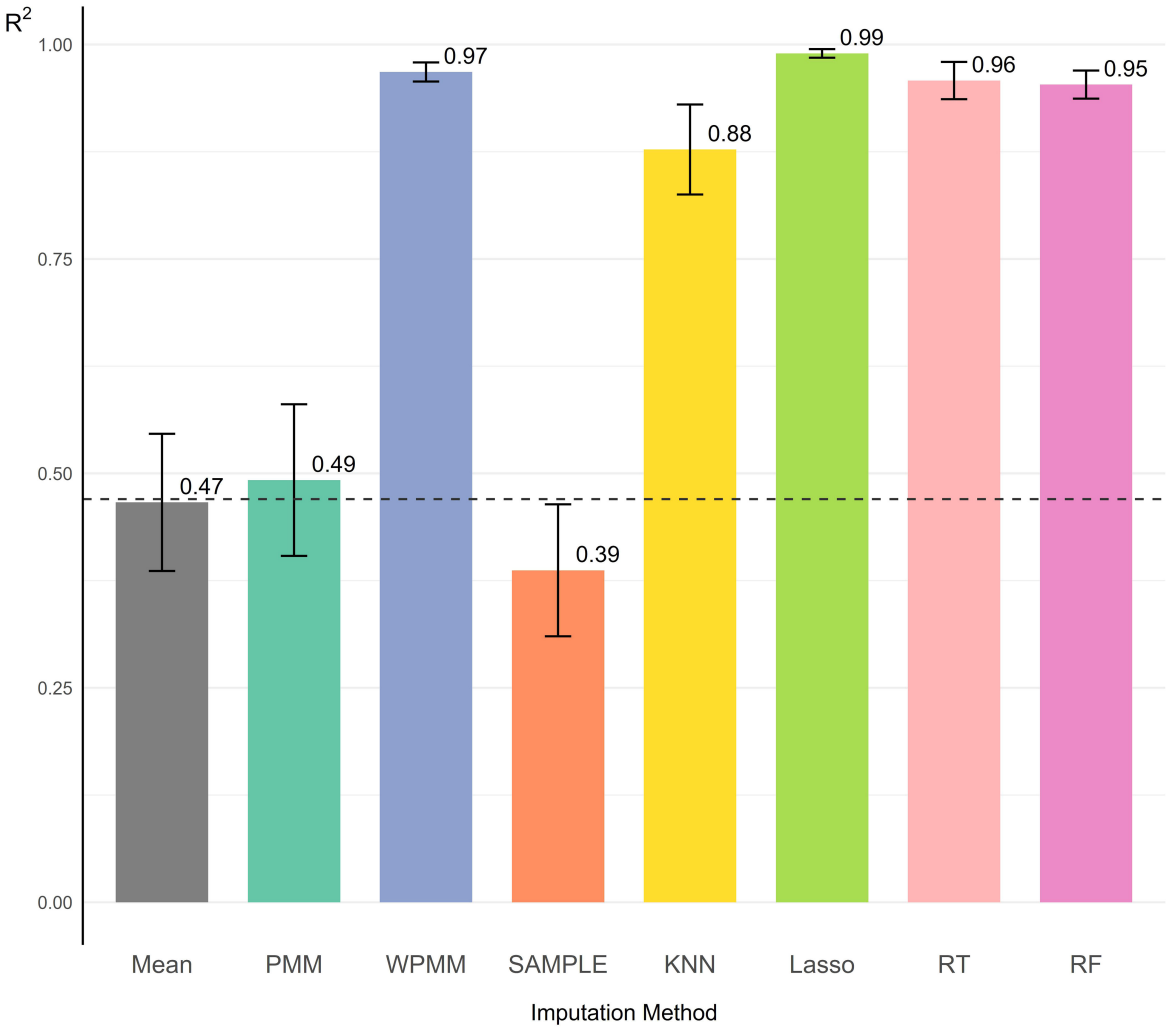
The coefficient of determination (R²) for the eight imputation models using the Bootstrap Evaluation for Structural Missingness Imputation (BESMI) on the partially overlapping genetic distance matrix ***M***. Each bar represents an imputation method: Predictive Mean Matching (PMM), Weighted Predictive Mean Matching (WPMM), random sampling (SAMPLE), K-Nearest Neighbours (KNN), Lasso regression (Lasso), Regression Trees (RT), Random Forest (RF), and mean. The height of each bar indicates the mean R² value across multiple BESMI runs, while the error bars represent standard deviations. The horizontal line marks the baseline (Mean) performance.

Eight imputation methods were explored and evaluated through BESMI on M (**Figure 4**). Lasso regression emerged as the best-performing method, with an $R^{2}$ of 0.99 ± 0.005 standard deviations, followed by WPMM (0.97 ± 0.011), RT (0.96 ± 0.022), RF (0.95 ± 0.016), and KNN (0.88 ± 0.052). The mean imputation (baseline) achieved an ${\text{R}}^{2}$ of 0.47 ± 0.080, while PMM performed similarly (0.49 ± 0.088). SAMPLE showed the lowest performance, with ${\text{R}}^{2}$ values below the baseline. Given its highest ${\text{R}}^{2}$ , Lasso regression was selected for imputing the structural missingness in M . Details about the merged matrix before ( M ) and after ( $M^{*}$ ) imputation are available in **Supplementary File 5**.

### 3.5 Estimation of DMY for potential host–endophyte combinations

Three sets of LMMs (LMM0, LMM1, and LMM2) were conducted to assess the impact of *endophytes* on the DMY of ryegrasses. Wald tests (**Table 1**) confirmed that the best-fitting models in each set adequately accounted for the main environmental (*Trial*) and temporal (*Trial : Harvest*) effects. The inclusion of *endophyte* effects was significant with *p* < 0.001 (Wald statistic = 36, *df* = 9).

**Table 1:** Wald tests of the best-fitting linear mixed models for DMY analysis.

Models	Fixed components	DF	Wald statistic	*p*-Value
LMM0	(*Intercept*)	1	46,923	< 0.001^***^
	*Trial*	17	15,815	< 0.001^***^
	*Trial*:*Harvest*	367	78,801	< 0.001^***^
	*Trial*:*Harvest*:lin (*Row*)	385	1,928	< 0.001^***^
	*Trial*:*Harvest*:lin (*Column*)	385	1,487	< 0.001^***^
LMM1	(*Intercept*)	1	29,281	< 0.001^***^
	*Trial*	17	16,267	< 0.001^***^
	*Trial*:*Harvest*	367	78,810	< 0.001^***^
	*Trial*:*Harvest*:lin (*Row*)	385	1,911	< 0.001^***^
	*Trial*:*Harvest*:lin (*Column*)	385	1,481	< 0.001^***^
LMM2	(*Intercept*)	1	39,812	< 0.001^***^
	*Trial*	17	30,008	< 0.001^***^
	*Endophyte*	9	36	< 0.001^***^
	*Trial*:*Harvest*	367	101,505	< 0.001^***^
	*Trial*:*Harvest*:lin (*Row*)	385	2,180	< 0.001^***^
	*Trial*:*Harvest*:lin (*Column*)	385	1,843	< 0.001^***^

DF, degree of freedom. *** means p< 0.001; the values are already in the table. No additional edit needed here.

All identified models (**Table 2**) consistently retained an order-1 autoregression structure for *Row* and *Column* in the residual components. The optimal structure for *Harvest* varied between models, featuring order-2 autoregression in the random components and either order-1 or order-2 autoregression in the residual components.

**Table 2:** Comparison of the best-fitting linear mixed models for DMY analysis.

Models	Y	Components	LogREML	AIC	MSE
LMM0	Yield	Xβ:∼ *Trial*+*Trial*:*Harvest*+*Trial*:*Harvest*:(lin(*Row*)+lin(*Column*)) Var(u):∼ fa(*Trial*,3):*Cultivar*+at(*Trial*):ar1(*Harvest*):*Cultivar* Var(ϵ):∼ at(*Trial*):ar3(*Harvest*):ar1(*Row*):ar1(*Column*)	− 236,106	472,602.6	83,007.68
LMM1	Yield	Xβ:∼ *Trial*+*Trial*:*Harvest*+*Trial*:*Harvest*:(lin(*Row*)+lin(*Column*)) Var(u):∼ fa(*Trial*,3):*Cultivar*+at(*Trial*):ar2(*Harvest*):*Cultivar*+*Endophyte* Var(ϵ):∼ at(*Trial*):ar2(*Harvest*):ar1(*Row*):ar1(*Column*)	− 236,075	472,537.2	83,310.92
LMM2	Yield	Xβ:∼ *Trial*+*Trial*:*Harvest*+*Trial*:*Harvest*:(lin(*Row*)+lin(*Column*))+*Endophyte* Var(u):∼ fa(*Trial*,3):*Cultivar*+at(*Trial*):ar2(*Harvest*):*Cultivar* Var(ϵ):∼ at(*Trial*):ar1(*Harvest*):ar1(*Row*):ar1(*Column*)	− 228,881	459,519.6	82,037.28

LogREML, the natural logarithm of restricted maximum likelihood; AIC, Akaike information criteria; MSE, mean squared error.

The identified LMM0 contained an order-3 factor analytic structure to account for genetic variances across environments and an order-1 autoregression variance structure for *Harvest* within *Trial*. The identified LMM1, which included *endophyte* as a random component, demonstrated improvement over LMM0, with a lower AIC (472,537.2 vs 472,602.6) and a Chi-square statistic of $\chi_{\left(12\right)}^{2}$ = 63.3, *p* < 0.001. Notably, genotypic variances accounted for 24.3% of the total estimated variance (26,332.7/108,375.3 × 100%), while *endophyte* explained 5.6% of the total estimated variance (6,061.8/108,375.3 × 100%). The identified LMM2, which modelled *endophyte* effects as fixed effects, further reduced the AIC to 459,519.6 and demonstrated the highest overall fit (AIC: 459,519.6, MSE = 82,037.28). Wherein, the genotypic variance component accounts for 25.3% of the total estimated variances (26,907.2/106,352.8 × 100%). Since REML models do not naturally estimate the variance of fixed effects, the variance explained by *endophyte* in LMM2 was calculated as the difference between the residual variances of the full model (LMM2) and its reduced version omitting the fixed *endophyte* component, accounting for 10.4% of total estimated variances (11,072.4/10,6352.8×100%).

**Figure 5:**
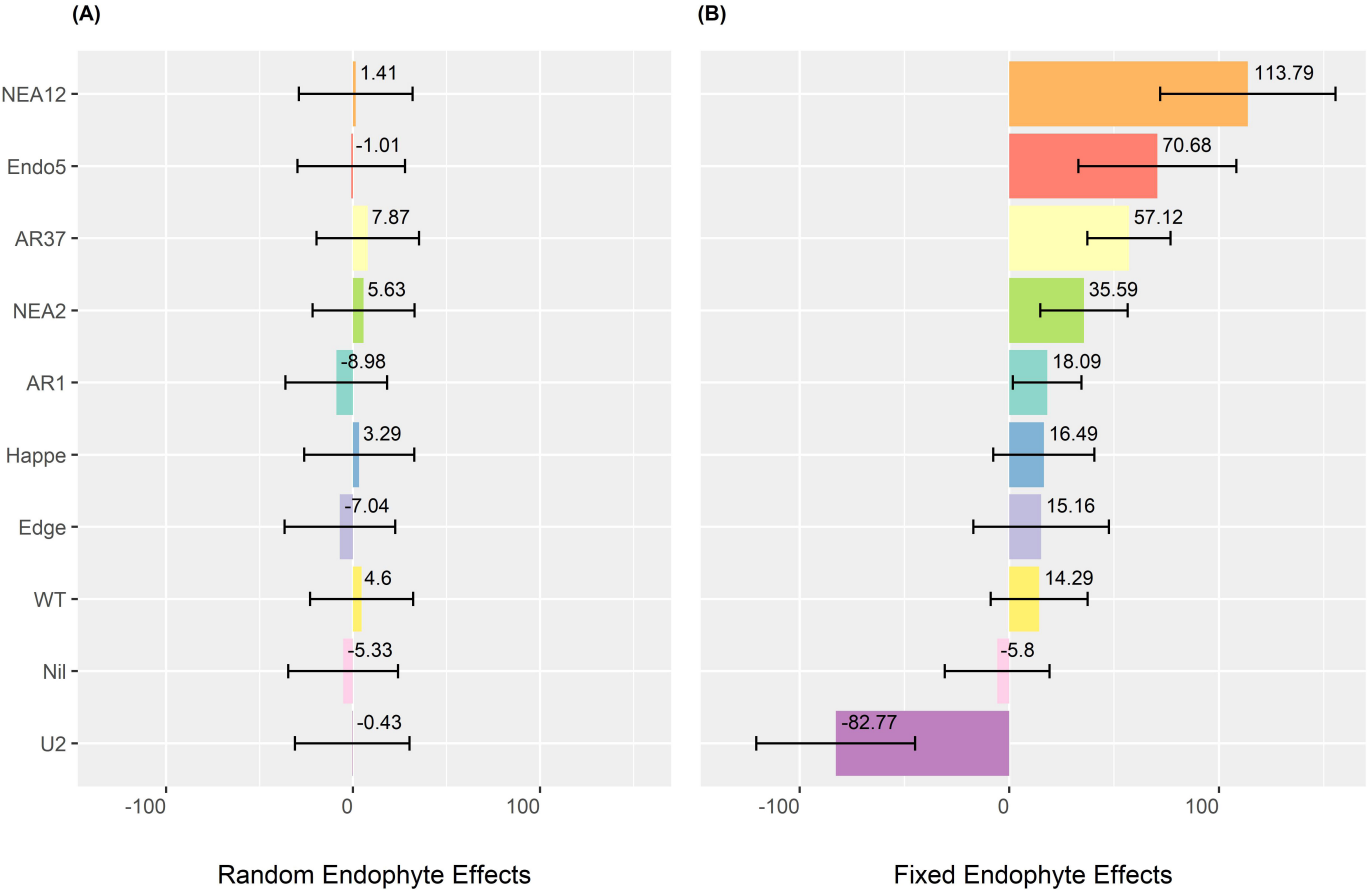
Estimated endophyte effects on dry matter yield (DMY) of ryegrass. **(A)** Random endophyte effects on DMY (kg DM/ha per harvest). **(B)** Fixed endophyte effects on DMY (kg DM/ha per harvest). Error bars indicate standard errors. Endophyte strains are arranged from highest to lowest effect based on their fixed effects. Nil means low or no endophytes.

**Figure 5** compares the *endophyte* effects as fixed versus random components. **Figure 5A** presents the random endophyte effects, which are compressed toward 0, reducing variation among endophytes. In contrast, **Figure 5B** illustrates endophyte effects as fixed components, revealing greater distinctions. Notably, NEA12 and *Epichloë uncinatum* (U2) exhibit the most significant positive and negative impacts on DMY, with effects of 113.79 kg DM/ha per harvest and − 82.77 kg DM/ha per harvest, respectively.

### 3.6 Phylogenetic–phenotypic association of DMY

The predicted population DMYs (kg DM/ha per harvest) derived from the identified LMM2 are provided in **Supplementary File 6**. This file also includes group estimates for genetically similar populations, genetic distances within each group, and estimates of potential host–endophyte combinations.

**Figure 6:**
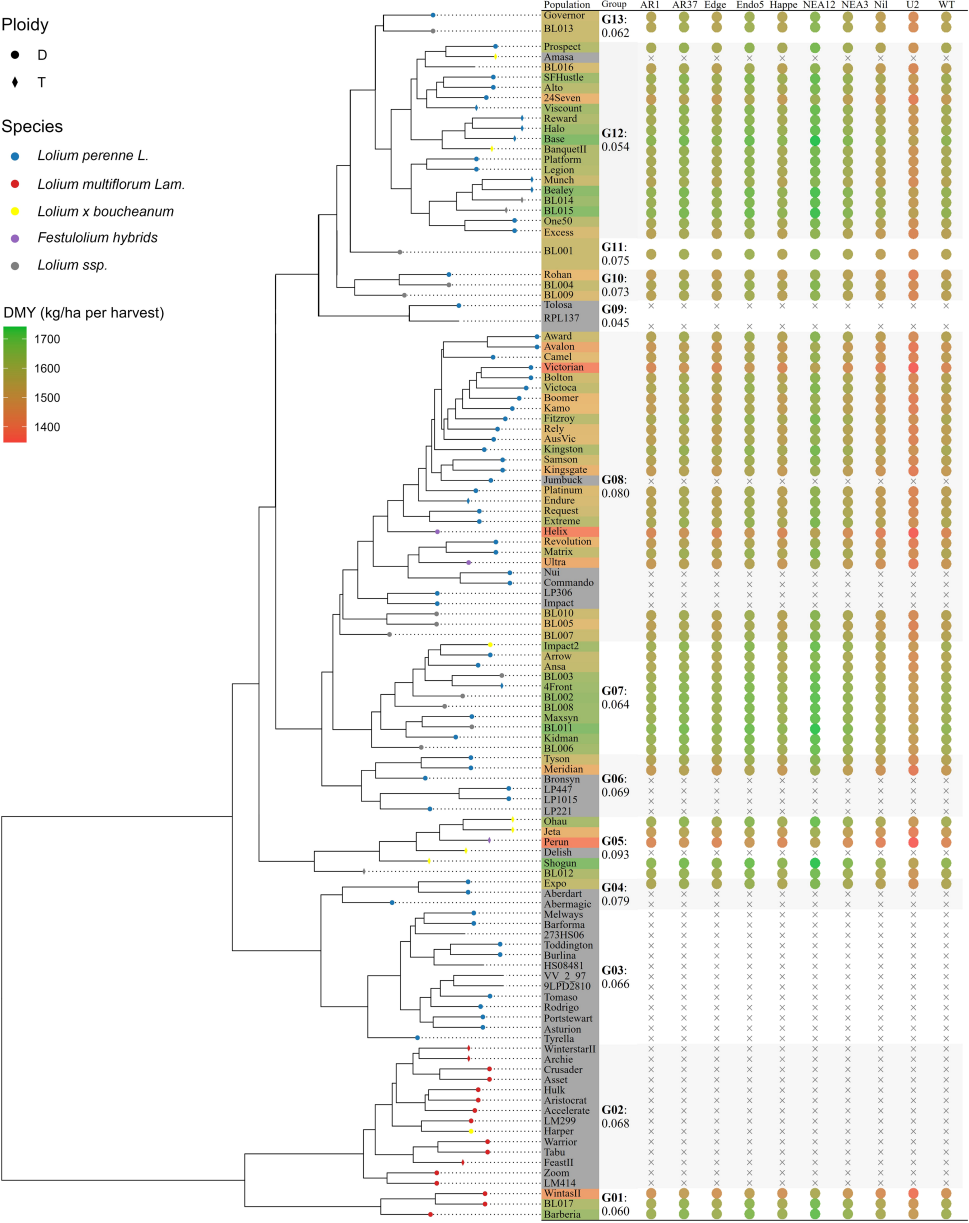
The phylogenetic tree of 113 ryegrass populations and its association with dry matter yield (DMY). Circles represent diploidy (D), while diamonds indicate tetraploidy (T) populations. Subspecies are colour-coded: *Lolium perenne* L. (blue), *Lolium multiflorum* Lam. (red), *Lolium x boucheanum* (yellow), Festulolium hybrids (purple), and *Lolium* ssp. (grey). The coloured table by the side illustrates the predicted DMYs (kg DM/ha per harvest). Group distances among populations are indicated, and the dot heatmap visualizes estimated DMYs for potential host–endophyte combinations.

**Figure 6** visualised the phylogenetic tree of the 113 ryegrass populations and its association with DMY predictions (kg DM/ha per harvest). The tree displays genetic distances ranging from 0 to 0.251, revealing genetically distinct ryegrass clades. In the upper section, a clade containing populations such as Prospect, Viscount Halo, Base, Bealey, and One50 exhibits high DMY performance, as indicated by the green colouration in the adjacent heatmap. This high-performing group has an estimated DMY of 1,575.2 kg DM/ha per harvest, with a standard error of 41.8 kg DM/ha per harvest and a genetic distance of 0.054 within the group. Another high-performing clade (DMY: 1,579.1 ± 45.2; genetic distances: 0–0.064) was observed in the middle section of the tree, comprising populations like Impact2, 4Front, Maxsyn, and Kidman. In contrast, the upper-middle section of the tree contains a clade (distance = 0.080) with populations ranging from Award and Avalon to BL007, consistently exhibiting lower DMY performance (1,507.8 ± 51.9), as indicated by the orange and red colouration. ANOVA revealed significant differences in DMY estimates across groups (*F*(9, 61) = 3.952, *p* < 0.001).

## 4 Discussion

### 4.1 Population genotyping

Ryegrass exhibits greater heterozygosity than other mainstay crops and exists in both diploid and tetraploid cultivars, resulting in a heterogeneous distribution of genetic variants within populations. This study found that the conventional discrete format inadequately encodes such variation, making genotyping in allele frequency format a more effective approach.

Ryegrass cultivars with similar genetic backgrounds but different endophytes clustered closely (**Figure 2**). This pattern is expected, as seedlings from base cultivars are typically inoculated with novel endophyte strains to maintain genetic consistency. However, a threshold value (0.0182) posed challenges in detecting subtle genetic differences between cultivars, possibly due to sequencing, genotyping, or data-processing protocols. Examples include Avalon and Award, as well as the trio of Victorian, Bolton, and Victoca. Interestingly, all these cultivars were developed through Australian ryegrass breeding programs, which focus on selecting broadly adapted yet genetically diverse cultivars from a Victorian ecotype. In many cases, the exact breeding programs are not documented, but they were largely based on an initial collection of plants from diverse established pastures. The multisite, half-sib family evaluation process used to develop Avalon perennial ryegrass was described by Croft et al. (2000).

In addition, population genotyping accurately captured the heterogeneous heterozygosity of ryegrass. The validation (**Supplementary File 2**) confirmed that when using a target capture assay, population genotyping can reliably differentiate cultivars with distinct genetic backgrounds. Specifically, bulking a minimum of 20 individuals per population demonstrated consistency with traditional individual genotyping methods, ensuring an accurate representation of ryegrass genomic variation. Further analysis showed that increasing the bulked sample size to at least 50 individuals provided a more conservative estimate of genetic variability within a population. This suggests a potential standard for future ryegrass studies. However, given the varying genetic diversity across plant species, researchers are encouraged to optimise bulk settings tailored to specific study requirements.

### 4.2 Multidimensional scaling alignment via Procrustes transformation

This study introduces an innovative approach to integrating diverse genetic datasets derived from different genotyping methods. While GDMs from independent studies capture valid internal relationships, they exhibit inconsistencies when directly compared, primarily due to differences in genotyping techniques and unique population entries across studies.

MDS alignment via Procrustes transformation was proposed to address this challenge. Each GDM was treated as a high-dimensional snapshot of true genetic relationships among tested populations. This assumption guided the development of an MDS-based calibration framework, which projects genetic distance data into computationally manageable lower-dimensional spaces, followed by Procrustes transformation. This approach calibrates inconsistencies across studies using shared populations as reference points while preserving internal genetic patterns. Notably, mapping the Procrustes-transformed configurations back to genetic distances resulted in a 73.2% reduction in SSD between datasets, demonstrating substantial bias reduction.

This method enables the integration of genetic data from diverse sources, allowing comparative analyses across studies with different sequencing approaches, SNP loci variations, or heterogeneous patterns of structural missingness. However, calibration performance is case-specific and depends on factors such as the number and representativeness of shared populations and the optimal dimensionality for transformation. To support further investigation, we have shared the source code (Zhu, 2025; **Supplementary File 3**) for researchers interested in applying this approach to their own datasets.

### 4.3 BESMI: an evaluation strategy for structured missingness imputation

Structural missingness often arises when merging genetic datasets from incompatible studies, leading to partially overlapping matrices in the merged dataset. The imputation of these missing values was framed as multiple propagated regression problems that leverage existing distance patterns in the data. BESMI was developed to assess imputation performance specifically for structural missingness—a scenario where traditional random cross-validation fails due to its inability to capture inherent data structures. By introducing missing values at the row/column level, BESMI effectively stimulates realistic structural missingness scenarios while preserving genetic structure integrity.

BESMI exhibited varying performance across imputation functions. Lasso regression generally outperformed others due to its selective consideration of population configurations via L1 regularisation. Similarly, methods that prioritise specific populations during prediction (WPMM, KNN, RF, RT) performed well—WPMM assigned variable weights to populations, while KNN leveraged the nearest populations for prediction. Conversely, methods treating all populations as predictors (PMM, SAMPLE) showed suboptimal performance. These findings underscore the importance of capturing inherent patterns in the genetic matrix when imputing structurally missing data.

The simulation study further confirmed that inherent patterns influence imputation performance. The best-performing model varied across simulated datasets with different genetic structures, highlighting the case-specific nature of structural missingness imputation. While Lasso regression performed well in our analysis, researchers are advised to apply BESMI to evaluate imputation functions on their own datasets to determine the most suitable approach. Additionally, the optimal number of iterations differed across datasets, underscoring the need to use BESMI in varied cases. The source code is provided for researchers interested in adapting BESMI to diverse study scenarios (Zhu, 2025; **Supplementary File 3**).

### 4.4 Phylogenetic analysis and breeding history

Ryegrass genetic analysis presents challenges due to the lack of definitive breeding histories. The merged GDM in this study provides extended quantitative measurements of genetic divergence among ryegrass populations. The phylogenetic tree generated from this extended GDM improves the resolution of genetic relationships among base ryegrass cultivars, aligning with previous findings by Pembleton et al. (2016). This enhanced analysis identifies four principal breeding clades: Italian ryegrass (G01 and G02, genetic distance: 0–0.128), European perennial ryegrass (G03: 0–0.066), Boucheanum hybrids (G05: 0–0.093), and Australian/New Zealand perennial ryegrass (G06, G07, and G08: 0–0.149). Importantly, these genetic findings challenge traditional classification methods. Cultivars such as BanquetII, Impact2, and Amasa—previously classified as Boucheanum hybrids based on morphological traits like awning—were genetically more aligned with perennial ryegrass. This discrepancy underscores the limitations of relying solely on morphological characteristics for species classification while demonstrating how extended phylogenetic analysis provides valuable insights into the genetic structures and breeding histories of commercial ryegrass cultivars.

### 4.5 Phylogenetic–phenotypic association

The phylogenetic–phenotypic association study offered valuable insight into ryegrass breeding programs. Current breeding efforts primarily rely on genomic prediction (GP) to estimate phenotypic performance, typically assuming a genetically coherent population to capture genetic variation and maintain stable linkage disequilibrium (Crossa et al., 2017; Daetwyler et al., 2012; Goddard, 2008; Habier et al., 2007; Isik et al., 2017; Meuwissen et al., 2001; Newell and Jannink, 2014; Shi et al., 2021; VanRaden, 2008). However, this assumption is often violated in populations with diverse genetic backgrounds, as estimates of SNP effects vary across populations (Alemu et al., 2024; Daetwyler et al., 2012; Goddard, 2008; Habier et al., 2007; Resende et al., 2012).

In contrast, the phylogenetic–phenotypic association approach leverages population relationships to assess breeding potential. Unlike GP, which relies on less interpretable marker estimates, this method provides more straightforward inferences by directly linking population relationships to performance variations within phylogenetic clades. For example, the perennial ryegrass Amasa is expected to exhibit high DMY performance due to its position within a high-performing clade (**Figure 5**), demonstrating the feasibility of evaluating the performance of closely related populations even in the absence of phenotypic data.

However, phenotypic data remain essential for validating inferences about desired traits. In certain groups, such as those spanning from Melways to Tyrella and from Winterstar II to LM414, the absence of field trial data completely prevents DMY estimation. Additionally, phylogenetic-based inferences require refinement in estimation precision, as they assume that closely clustered cultivars exhibit similar phenotypic characteristics. Compared with GP-based estimation, which directly fits genetic correlations, this approach provides a more precise means of accounting for phenotypic variation (Arojju et al., 2020, 2018; Cericola et al., 2018; Endelman, 2011; Esfandyari et al., 2020; Faville et al., 2021, 2018, 2016; Fè et al., 2016, 2015; Grinberg et al., 2016; Jahufer et al., 2021; Keep et al., 2020; Konkolewska et al., 2023; Lin et al., 2016; Malmberg et al., 2023).

Moreover, phylogenetic-based inferences would be particularly beneficial when GP identified multiple candidates with similar genetically estimated breeding values in a selection process. Breeders could choose germplasm within related clades to maintain performance consistency or select germplasm from distant clades to introduce genetic variability, depending on whether the breeding goal is to stabilise specific traits or develop cultivars adaptable to a broader environment. Furthermore, the phylogenetic analysis helps breeders select germplasm with similar genetic components without relying on external sources, preserving proprietary genetic resources while enhancing trait performance.

Given these considerations, the phylogenetic–phenotypic association method was proposed as a complementary approach to GP, enhancing breeding decisions for complex plant species like ryegrass. This method not only addresses GP’s limitations by leveraging population structures but also provides a more interpretable insight into breeding potential and genetic diversity.

### 4.6 Endophyte impacts on ryegrass DMY estimates

Estimating DMY in ryegrass was challenged by the presence of endophytes, as genetically similar ryegrass cultivars often exhibited varying DMY responses to specific endophytes. This challenge was addressed by differentiating endophyte effects, with statistical modelling (LMM2) confirming their significance. Treating endophytes as fixed effects provided clearer differentiation (**Figure 5**), suggesting that assuming consistent endophyte effects across base ryegrass cultivars could effectively capture their impact on host plant DMY.

The impact of endophytes may be attributed to their distinct alkaloid profiles and compatibility with host plants (Eady, 2021; Popay and Hume, 2013; Vassiliadis et al., 2023). NEA12 and AR37 positively influence DMY, likely due to their production of epoxy-janthitrems, which enhance the host plant’s pest resistance (Vassiliadis et al., 2023). The distribution of alkaloids within the plant may also affect DMY performance. Vassiliadis et al. (2023) found that janthitrem is evenly distributed between shoots and roots, whereas peramine—another alkaloid commonly found in AR1, NEA2, and Edge—is concentrated almost entirely in the shoots. This more even distribution of janthitrem may provide enhanced protection against both above- and belowground pests compared to peramine. In contrast, U2 produces lolines, which, although beneficial against pests, may impose a metabolic cost. Additionally, U2 is a nonnative endophyte for ryegrasses and was originally associated with meadow fescue. This may explain its reduced compatibility with ryegrass hosts and the lack of growth benefits observed in other native host–endophyte combinations (Eady, 2021).

The initial assessment of endophyte impacts in this study also reinforced insights into the phylogenetic–DMY association and enabled a comprehensive estimation of DMY for untested host–endophyte combinations. This information can help companies assess how endophytes from external sources may affect the performance of their own cultivars. Future research could benefit from developing methods to biologically separate and recombine endophytes with plants, as well as implementing more balanced tests to improve the understanding of endophyte–ryegrass interactions in DMY estimates. However, it is important to acknowledge the limitations posed by the imbalanced dataset used in this study. Not all endophytes were present or tested across every ryegrass, primarily because endophytes were inherently confounded with host cultivar(s) due to commercial restrictions between endophyte owners and ryegrass breeding companies. This limitation has hindered comprehensive evaluations of endophyte impacts across a broader range of ryegrass cultivars.

### 4.7 Applications beyond ryegrass

While this study focused on ryegrass, the approaches presented have potential applications across a wide range of plant species, particularly those with complex genetic backgrounds. The population sequencing approach could be adapted for other forage species, such as lucerne or tall fescue. The MDS-based calibration provides valuable solutions for integrating datasets from incompatible platforms, while BESMI offers an effective strategy for addressing structural missingness in genetic data. Notably, combining these methods for dataset integration could enhance cross-institute collaboration, particularly in cases where raw data are unavailable due to permission constraints or when data originate from incompatible protocols. Addressing these discrepancies is essential for integrating inferences from independent studies and expanding their applications without requiring large-scale *de novo* sequencing or genome assembly. Furthermore, phylogenetic–phenotypic association can serve as a supplementary approach for assessing species with heterogeneous genetic backgrounds in breeding programs. This approach provides researchers and breeders with clearer insights, aiding in the preservation of genetic diversity. Moreover, these insights can enhance the communication of scientific findings to a broader range of agricultural stakeholders.

## Data Availability

The datasets presented in this study can be found in online repositories. The names of the repository/repositories and accession number(s) can be found in the article/**Supplementary Material**.

## Supplementary material

Supplementary File 1 Population_Sequencing_Details.

Supplementary File 2 Population_Genotyping_Validation.

Supplementary File 3 Integration_of_Partially_Overlapped_GDMs.

Supplementary File 4 Information_of_the_2_Genetic_Datasets.

Supplementary File 5 M.

Supplementary File 6 DMY_Predictions.

## References

[B1] Alemu A. Åstrand J. Montesinos-López O. Sánchez J. Fernández-Gónzalez J. Tadesse W.. (2024). Genomic selection in plant breeding: Key factors shaping two decades of progress. Mol. Plant 17, 552–578. doi: 10.1016/j.molp.2024.03.007 38475993

[B2] Andersen J. R. Jensen L. B. Asp T. Lübberstedt T. (2006). Vernalization response in perennial ryegrass (Lolium perenne L.) involves orthologues of diploid wheat (Triticum monococcum) VRN1 and rice (Oryza sativa) hd1. Plant Mol. Biol. 60, 481–494. doi: 10.1007/s11103-005-4815-1 16525886

[B3] Andreella A. De Santis R. Vesely A. Finos L. (2023). Procrustes-based distances for exploring between-matrices similarity. Stat. Methods Appl. 32, 867–882. doi: 10.1007/s10260-023-00689-y

[B4] Arojju S. K. Cao M. Trolove M. Barrett B. A. Inch C. Eady C.. (2020). Multi-trait genomic prediction improves predictive ability for dry matter yield and water-soluble carbohydrates in perennial ryegrass. Front. Plant Sci. 11. doi: 10.3389/fpls.2020.01197 PMC7426495 32849742

[B5] Arojju S. K. Conaghan P. Barth S. Milbourne D. Casler M. D. Hodkinson T. R.. (2018). Genomic prediction of crown rust resistance in Lolium perenne. BMC Genet. 19, 35. doi: 10.1186/s12863-018-0613-z 29843601 PMC5975627

[B6] Barre P. Asp T. Byrne S. Casler M. Faville M. Rognli O. A.. (2022). “Genomic prediction of complex traitsComplex traits in forage plants species: perennial grasses case,” in Genomic Prediction of Complex Traits: Methods and Protocols. Eds. Ahmadi N. Bartholomé J. (Springer US, New York, NY), 521–541. doi: 10.1007/978-1-0716-2205-6_19 35451789

[B7] Blackmore T. Thorogood D. Skøt L. McMahon R. Powell W. Hegarty M. Germplasm dynamics: the role of ecotypic diversity in shaping the patterns of genetic variation in Lolium perenne. Scientific Reports 6, 22603. doi: 10.1038/srep22603 PMC4776279 26935901

[B8] Bhattacharjee A. Bayzid M. S. (2020). Machine learning based imputation techniques for estimating phylogenetic trees from incomplete distance matrices. BMC Genomics 21, 497. doi: 10.1186/s12864-020-06892-5 32689946 PMC7370488

[B9] Borg I. Groenen P. J. (2005). Modern multidimensional scaling: Theory and applications (New York, United States: Springer Science & Business Media). doi: 10.1007/0-387-28981-X

[B10] Bozdogan H. (1987). Model selection and Akaike’s Information Criterion (AIC): The general theory and its analytical extensions. Psychometrika 52, 345–370. doi: 10.1007/BF02294361

[B11] Browning B. L. Browning S. R. (2016). Genotype imputation with millions of reference samples. Am. J. Hum. Genet. 98, 116–126. doi: 10.1016/j.ajhg.2015.11.020 26748515 PMC4716681

[B12] Browning B. L. Tian X. Zhou Y. Browning S. R. (2021). Fast two-stage phasing of large-scale sequence data. Am. J. Hum. Genet. 108, 1880–1890. doi: 10.1016/j.ajhg.2021.08.005 34478634 PMC8551421

[B13] Butler D. Cullis B. R. Gilmour A. R. Gogel B. J. Thompson R. (2009). ASReml-R reference manual, release 3 (Hemel Hempstead, UK: VSN International Ltd). Available at: https://asreml.kb.vsni.co.uk/wp-content/uploads/sites/3/2018/02/ASReml-3-User-Guide.pdf (Accessed November 1, 2024).

[B14] Cericola F. Lenk I. Fè D. Byrne S. Jensen C. S. Pedersen M. G.. (2018). Optimized use of low-depth genotyping-by-sequencing for genomic prediction among multi-parental family pools and single plants in perennial ryegrass (Lolium perenne L.). Front. Plant Sci. 9. doi: 10.3389/fpls.2018.00369 PMC5871745 29619038

[B15] Chapman D. F. Bryant J. R. Olayemi M. E. Edwards G. R. Thorrold B. S. McMillan W. H.. (2017). An economically based evaluation index for perennial and short-term ryegrasses in New Zealand dairy farm systems. Grass Forage Sci. 72, 1–21. doi: 10.1111/gfs.12213

[B16] Chapman D. F. Edwards G. R. Stewart A. V. McEvoy M. O’Donovan M. Waghorn G. C. (2015). Valuing forages for genetic selection: what traits should we focus on? Anim. Production Sci. 55, 869–882. doi: 10.1071/AN14838

[B17] Chen D. Fan B. Oliver C. Borgwardt K. (2023). Unsupervised manifold alignment with joint multidimensional scaling. arXiv preprint arXiv:2207.02968. doi: 10.48550/arXiv.2207.02968

[B18] Chen Z. Jin Y. Yao X. Chen T. Wei X. Li C.. (2020). Fungal endophyte improves survival of lolium perenne in low fertility soils by increasing root growth, metabolic activity and absorption of nutrients. Plant Soil 452, 185–206. doi: 10.1007/s11104-020-04556-7

[B19] Chen G. Wang X. Sun Q. Tang Z.-Z. (2025). Multidimensional scaling improves distance-based clustering for microbiome data. Bioinformatics 41 (2). doi: 10.1093/bioinformatics/btaf042 PMC11814494 39874446

[B20] Croft V. M. Cunningham P. J. Anderson M. W. Smith K. F. Reed K. F. M. (2000). Register of Australian herbage plant cultivars. Lolium perenne cv. *Avalon* . Aust. J. Exp. Agric. 40, 1199–1200. doi: 10.1071/EA00085_CU

[B21] Crossa J. Pérez-Rodríguez P. Cuevas J. Montesinos-López O. A. Jarquín D. Campos G.. (2017). Genomic selection in plant breeding: methods, models, and perspectives. Trends Plant Sci. 22, 961–975. doi: 10.1016/j.tplants.2017.08.011 28965742

[B22] Daetwyler H. D. Kemper K. E. van der Werf J. H. J. Hayes B. J. (2012). Components of the accuracy of genomic prediction in a multi-breed sheep population1. J. Anim. Sci. 90, 3375–3384. doi: 10.2527/jas.2011-4557 23038744

[B23] Danecek P. Bonfield J. K. Liddle J. Marshall J. Ohan V. Pollard M. O.. (2021). Twelve years of SAMtools and BCFtools. GigaScience 10 (2). doi: 10.1093/gigascience/giab008 PMC7931819 33590861

[B24] Devi R. Verma R. Dhalaria R. Kumar A. Kumar D. Puri S.. (2023). A systematic review on endophytic fungi and its role in the commercial applications. Planta 257, 70. doi: 10.1007/s00425-023-04087-2 36856911

[B25] Du W. Wang X. Zhao X. Pei Y. Xia L. Zhao Q.. (2024). How high-throughput sequencing empowers the research of polyploidy in vegetable crops. Vegetable Res. 4, e006. doi: 10.48130/vegres-0024-0005

[B26] Eady C. (2021). The impact of alkaloid-producing epichloë Endophyte on forage ryegrass breeding: A New Zealand perspective. Toxins 13, 158. doi: 10.3390/toxins13020158 33670470 PMC7922046

[B27] Elshire R. J. Glaubitz J. C. Sun Q. Poland J. A. Kawamoto K. Buckler E. S.. (2011). A robust, simple genotyping-by-sequencing (GBS) approach for high diversity species. PloS One 6, e19379. doi: 10.1371/journal.pone.0019379 21573248 PMC3087801

[B28] Endelman J. B. (2011). Ridge regression and other kernels for genomic selection with R package rrBLUP. Plant Genome 4 (3). doi: 10.3835/plantgenome2011.08.0024

[B29] Esfandyari H. Fè D. Tessema B. B. Janss L. L. G. Jensen J. (2020). Effects of different strategies for exploiting genomic selection in perennial ryegrass breeding programs. G3 Genes|genome|genetics 10, 3783–3795. doi: 10.1534/g3.120.401382 PMC7534426 32819970

[B30] Esqueda M. Yen A. L. Rochfort S. Guthridge K. M. Powell K. S. Edwards J.. (2017). A review of perennial ryegrass endophytes and their potential use in the management of African black beetle in perennial grazing systems in Australia. Front. Plant Sci. 8. doi: 10.3389/fpls.2017.00003 PMC5244474 28154571

[B31] Faville M. J. Ganesh S. Cao M. Jahufer M. Z. Z. Bilton T. P. Easton H. S.. (2018). Predictive ability of genomic selection models in a multi-population perennial ryegrass training set using genotyping-by-sequencing. Theor. Appl. Genet. 131, 703–720. doi: 10.1007/s00122-017-3030-1 29264625 PMC5814531

[B32] Faville M. J. Ganesh S. Moraga R. Easton H. S. Jahufer M. Z. Z. Elshire R. E.. (2016). “Development of genomic selection for perennial ryegrass,” in Breeding in a World of Scarcity. Eds. Roldán-Ruiz I. Baert J. Reheul D., (Springer International Publishing, Cham), 139–143. doi: 10.1007/978-3-319-28932-8

[B33] Faville M. Schmidt J. Trolove M. Moran P. Hong W. Cao M.. (2021). Empirical assessment of a genomic breeding strategy in perennial ryegrass. J. New Z. Grasslands 83, 115–122. doi: 10.33584/jnzg.2021.83.3490

[B34] Fè D. Ashraf B. H. Pedersen M. G. Janss L. Byrne S. Roulund N.. (2016). Accuracy of genomic prediction in a commercial perennial ryegrass breeding program. Plant Genome 9 (3). doi: 10.3835/plantgenome2015.11.0110 27902790

[B35] Fè D. Cericola F. Byrne S. Lenk I. Ashraf B. Pedersen M. G.. (2015). Genomic dissection and prediction of heading date in perennial ryegrass. BMC Genomics 16 (1). doi: 10.1186/s12864-015-2163-3 PMC4642674 26559662

[B36] Frei D. Veekman E. Grogg D. Stoffel-Studer I. Morishima A. Shimizu-Inatsugi R.. (2021). Ultralong oxford nanopore reads enable the development of a reference-grade perennial ryegrass genome assembly. Genome Biol. Evol. 13 (8). doi: 10.1093/gbe/evab159 PMC8358221 34247248

[B37] Fu Y. B. (2015). Understanding crop genetic diversity under modern plant breeding. Theor. Appl. Genet. 128, 2131–2142. doi: 10.1007/s00122-015-2585-y 26246331 PMC4624815

[B38] Gilliland T. J. Ball T. Hennessy D. (2021). Opportunities and challenges for breeding perennial ryegrass cultivars with improved livestock production potential. Irish J. Agric. Food Res. 59 (2). doi: 10.15212/ijafr-2020-0111

[B39] Giri K. Chia K. Chandra S. Smith K. F. Leddin C. M. Ho C. K. M.. (2019). Modelling and prediction of dry matter yield of perennial ryegrass cultivars sown in multi-environment multi-harvest trials in south-eastern Australia. Field Crops Res. 243, 107614. doi: 10.1016/j.fcr.2019.107614

[B40] Goddard M. (2008). Genomic selection: Prediction of accuracy and maximisation of long term response. Genetica 136, 245–257. doi: 10.1007/s10709-008-9308-0 18704696

[B41] Gower J. C. (1966). Some distance properties of latent root and vector methods used in multivariate analysis. Biometrika 53, 325–338. doi: 10.1093/biomet/53.3-4.325

[B42] Gower J. C. (1975). Generalized procrustes analysis. Psychometrika 40, 33–51. doi: 10.1007/BF02291478

[B43] Grinberg N. F. Lovatt A. Hegarty M. Lovatt A. Skøt K. P. Kelly R.. (2016). Implementation of genomic prediction in lolium perenne (L.) breeding populations. Front. Plant Sci. 7. doi: 10.3389/fpls.2016.00133 PMC4751346 26904088

[B44] Guan X. Yuyama N. Stewart A. Ding C. Xu N. Kiyoshi T.. (2017). Genetic diversity and structure of lolium species surveyed on nuclear simple sequence repeat and cytoplasmic markers. Front. Plant Sci. 8. doi: 10.3389/fpls.2017.00584 PMC5399756 28484473

[B45] Guo X. Cericola F. Fè D. Pedersen M. G. Lenk I. Jensen C. S.. (2018). Genomic prediction in tetraploid ryegrass using allele frequencies based on genotyping by sequencing. Front. Plant Sci. 9. doi: 10.3389/fpls.2018.01165 PMC6104567 30158944

[B46] Habier D. Fernando R. L. Dekkers J. C. M. (2007). The impact of genetic relationship information on genome-assisted breeding values. Genetics 177, 2389–2397. doi: 10.1534/genetics.107.081190 18073436 PMC2219482

[B47] Hearn C. Egan M. Berry D. P. Geoghegan A. O’Leary M. Lynch M. B.. (2021). Agronomic performance of ten perennial ryegrass varieties on commercial grassland farms. J. Agric. Sci. 159, 604–614. doi: 10.1017/S0021859621000927

[B48] Howie B. Marchini J. Stephens M. (2011). Genotype imputation with thousands of genomes. G3 (Bethesda) 1, 457–470. doi: 10.1534/g3.111.001198 22384356 PMC3276165

[B49] Isik F. Holland J. Maltecca C. (2017). Genetic data analysis for plant and animal breeding. doi: 10.1007/978-3-319-55177-7

[B50] Jahufer M. Z. Z. Arojju S. K. Faville M. J. Ghamkhar K. Luo D. Arief V.. (2021). Deterministic and stochastic modelling of impacts from genomic selection and phenomics on genetic gain for perennial ryegrass dry matter yield. Sci. Rep. 11, 13265. doi: 10.1038/s41598-021-92537-w 34168203 PMC8225875

[B51] Jobin M. Schurz H. Henn B. M. (2018). IMPUTOR: phylogenetically aware software for imputation of errors in next-generation sequencing. Genome Biol. Evol. 10, 1248–1254. doi: 10.1093/gbe/evy088 29722813 PMC5961346

[B52] Keep T. Sampoux J.-P. Blanco-Pastor J. L. Dehmer K. J. Hegarty M. J. Ledauphin T.. (2020). High-throughput genome-wide genotyping to optimize the use of natural genetic resources in the grassland species perennial ryegrass (Lolium perenne L.). G3 Genes|Genomes|Genetics 10, 3347–3364. doi: 10.1534/g3.120.401491 32727925 PMC7466994

[B53] Knaus B. J. Grünwald N. J. (2017). VCFR: a package to manipulate and visualize variant call format data in R. Mol. Ecol. Resour. 17, 44–53. doi: 10.1111/1755-0998.12549 27401132

[B54] Konkolewska A. Phang S. Conaghan P. Milbourne D. Lawlor A. Byrne S. (2023). Genomic prediction of seasonal forage yield in perennial ryegrass. Grassland Res. 2, 167–181. doi: 10.1002/glr2.12058

[B55] Kruskal J. Wish M. (1978). Multidimensional scaling (Thousand Oaks, California: SAGE Publications, Inc.). doi: 10.4135/9781412985130

[B56] Leddin C. Giri K. Smith K. (2022). Variation in the nutritive characteristics of modern perennial ryegrass cultivars in south-eastern Australian dairy environments and prospects for inclusion in the Australian forage value index (FVI). Agronomy 12, 136. doi: 10.3390/agronomy12010136

[B57] Leddin C. Jacobs J. Smith K. Giri K. Malcolm B. Ho C. (2018). Development of a system to rank perennial ryegrass cultivars according to their economic value to dairy farm businesses in south-eastern Australia. Anim. Production Sci. 58. doi: 10.1071/AN17815

[B58] Lee M. A. Howard-Andrews V. Chester M. (2019). Resistance of multiple diploid and tetraploid perennial ryegrass (Lolium perenne L.) varieties to three projected drought scenarios for the UK in 2080. Agronomy 9, 159. doi: 10.3390/agronomy9030159

[B59] Li H. (2013). Aligning sequence reads, clone sequences and assembly contigs with BWA-MEM. arXiv: Genomics. doi: 10.48550/arXiv.1303.3997. Preprint.

[B60] Lin Z. Cogan N. O. I. Pembleton L. W. Spangenberg G. Forster J. W. Hayes B. J.. (2016). Genetic gain and inbreeding from genomic selection in a simulated commercial breeding program for perennial ryegrass. Plant Genome 9 (1). doi: 10.3835/plantgenome2015.06.0046 27898764

[B61] Malmberg M. M. Pembleton L. W. Baillie R. C. Drayton M. C. Sudheesh S. Kaur S.. (2018). Genotyping-by-sequencing through transcriptomics: implementation in a range of crop species with varying reproductive habits and ploidy levels. Plant Biotechnol. J. 16, 877–889. doi: 10.1111/pbi.12835 28913899 PMC5866951

[B62] Malmberg M. Smith C. Thakur P. Drayton M. Wilson J. Shinozuka M.. (2023). Developing an integrated genomic selection approach beyond biomass for varietal protection and nutritive traits in perennial ryegrass (Lolium perenne L.). Theor. Appl. Genet. 136. doi: 10.1007/s00122-023-04263-8 PMC10006259 36897387

[B63] Marchini J. Howie B. Myers S. McVean G. Donnelly P. (2007). A new multipoint method for genome-wide association studies by imputation of genotypes. Nat. Genet. 39, 906–913. doi: 10.1038/ng2088 17572673

[B64] Martin M. (2011). Cutadapt removes adapter sequences from high-throughput sequencing reads. EMBnet J. 17, 3. doi: 10.14806/ej.17.1.200

[B65] McEvoy M. O’Donovan M. Shalloo L. (2011). Development and application of an economic ranking index for perennial ryegrass cultivars. J. Dairy Sci. 94, 1627–1639. doi: 10.3168/jds.2010-3322 21338830

[B66] Merino V. Balocchi O. Rivero M. (2019). Milk production, milk quality, and behaviour of dairy cows grazing on swards with low and high water-soluble carbohydrates content in autumn: A pilot trial. Animals: an Open Access J. MDPI 9. doi: 10.3390/ani9121012 PMC6940813 31766428

[B67] Meuwissen T. H. Hayes B. J. Goddard M. E. (2001). Prediction of total genetic value using genome-wide dense marker maps. Genetics 157, 1819–1829. doi: 10.1093/genetics/157.4.1819 11290733 PMC1461589

[B68] Naito T. Okada Y. (2024). Genotype imputation methods for whole and complex genomic regions utilizing deep learning technology. J. Hum. Genet. 69, 481–486. doi: 10.1038/s10038-023-01213-6 38225263 PMC11422162

[B69] Nei M. (1972). Genetic distance between populations. Am. Nat. 106, 283–292. doi: 10.1086/282771

[B70] Newell M. A. Jannink J. L. (2014). Genomic selection in plant breeding(New York: Humana Press). 1145, 117–130. doi: 10.1007/978-1-4939-0446-4_10 24816664

[B71] Nguyen T. V. Bolormaa S. Reich C. M. Chamberlain A. J. Vander Jagt C. J. Daetwyler H. D.. (2024). Empirical versus estimated accuracy of imputation: optimising filtering thresholds for sequence imputation. Genet. Selection Evol. 56, 72. doi: 10.1186/s12711-024-00942-2 PMC11566673 39548370

[B72] Oksanen J. Simpson G. L. Blanchet F. G. Kindt R. Legendre P. Minchin P. R.. (2024). vegan: community ecology package. doi: 10.32614/CRAN.package.vegan

[B73] Paina C. Byrne S. Studer B. Rognli O. A. Asp T. (2016). Using a candidate gene-based genetic linkage map to identify QTL for winter survival in perennial ryegrass. PloS One 11, e0152004. doi: 10.1371/journal.pone.0152004 27010567 PMC4807000

[B74] Pasquali E. Palumbo F. Barcaccia G. (2022). Assessment of the genetic distinctiveness and uniformity of pre-basic seed stocks of Italian ryegrass varieties. Genes (Basel) 13 (11). doi: 10.3390/genes13112097 PMC9690090 36421771

[B75] Pedersen T. L. (2024). ggforce: accelerating ‘ggplot2’. Available online at: https://ggforce.data-imaginist.com (Accessed November 1, 2024).

[B76] Pembleton L. W. Cogan N. O. I. Forster J. W. (2013). StAMPP: an R package for calculation of genetic differentiation and structure of mixed-ploidy level populations. Mol. Ecol. Resour. 13, 946–952. doi: 10.1111/1755-0998.12129 23738873

[B77] Pembleton L. W. Drayton M. C. Bain M. Baillie R. C. Inch C. Spangenberg G. C.. (2016). Targeted genotyping-by-sequencing permits cost-effective identification and discrimination of pasture grass species and cultivars. Theor. Appl. Genet. 129, 991–1005. doi: 10.1007/s00122-016-2678-2 26883039

[B78] Pembleton L. W. Inch C. Baillie R. C. Drayton M. C. Thakur P. Ogaji Y. O. (2018). Exploitation of data from breeding programs supports rapid implementation of genomic selection for key agronomic traits in perennial ryegrass. Theor. Appl. Genet. 131, 1891–1902. doi: 10.1007/s00122-018-3121-7 29860624 PMC6096624

[B79] Peres-Neto P. R. Jackson D. A. (2001). How well do multivariate data sets match? The advantages of a Procrustean superimposition approach over the Mantel test. Oecologia 129, 169–178. doi: 10.1007/s004420100720 28547594

[B80] Popay A. J. Hume D. E. (2013).Endophytes for improving ryegrass performance: current status and future possibilities. Available online at: https://uknowledge.uky.edu/igc/22/2-13/7 (Accessed November 1, 2024).

[B81] R Core Team (2024). R: A language and Environment for Statistical Computing (Vienna, Austria: R Foundation for Statistical Computing). Available at: https://www.R-project.org/ (Accessed November 1, 2024).

[B82] Resende M. F. Jr. Muñoz P. Resende M. D. Garrick D. J. Fernando R. L. Davis J. M.. (2012). Accuracy of genomic selection methods in a standard data set of loblolly pine (Pinus taeda L.). Genetics 190, 1503–1510. doi: 10.1534/genetics.111.137026 22271763 PMC3316659

[B83] Robinson G. K. (1991). That BLUP is a good thing: the estimation of random effects. Stat. Sci. 6 15-32, 18. doi: 10.1214/ss/1177011926

[B84] Roshyara N. R. Scholz M. (2015). Impact of genetic similarity on imputation accuracy. BMC Genet. 16, 90. doi: 10.1186/s12863-015-0248-2 26193934 PMC4509609

[B85] Shi S. Li X. Fang L. Liu A. Su G. Zhang Y.. (2021). Genomic prediction using bayesian regression models with global–local prior. Front. Genet. 12. doi: 10.3389/fgene.2021.628205 PMC8083873 33936162

[B86] Torgerson W. S. (1952). Multidimensional scaling: I. Theory and method. Psychometrika 17, 401–419. doi: 10.1007/BF02288916 5217606

[B87] Torgo L. (2011). Data mining with R: learning with case studies. 1/Ed (New York: chapman and hall/CRC). doi: 10.1201/9780429292859

[B88] Van Buuren S. (2018). “Flexible imputation of missing data,” 2/Ed (New York: Chapman and Hall/CRC). doi: 10.1201/9780429492259

[B89] Van Buuren S. Groothuis-Oudshoorn K. (2011). mice: multivariate imputation by chained equations in R. J. Stat. Software 45, 1–67. doi: 10.18637/jss.v045.i03

[B90] Van der Auwera G. A. O’Connor B. D. (2020). Genomics in the cloud: using Docker, GATK, and WDL in Terra (Sebastopol, California, United States: O’Reilly Media).

[B91] VanRaden P. M. (2008). Efficient methods to compute genomic predictions. J. Dairy Sci. 91, 4414–4423. doi: 10.3168/jds.2007-0980 18946147

[B92] Vassiliadis S. Reddy P. Hemsworth J. Spangenberg G. C. Guthridge K. M. Rochfort S. J. (2023). Quantitation and distribution of epichloë-derived alkaloids in perennial ryegrass tissues. Metabolites 13, 205. doi: 10.3390/metabo13020205 36837825 PMC9966479

[B93] Wang C. Mahadevan S. (2008). “Manifold alignment using procrustes analysis,” in Proceedings of the 25th international conference on Machine learning. (Helsinki, Finland: Association for Computing Machinery (ACM)) 1120–1127. doi: 10.1145/1390156.1390297

[B94] Wang J. Pembleton L. W. Baillie R. C. Drayton M. C. Hand M. L. Bain M.. (2014). Development and implementation of a multiplexed single nucleotide polymorphism genotyping tool for differentiation of ryegrass species and cultivars. Mol. Breed. 33, 435–451. doi: 10.1007/s11032-013-9961-6

[B95] Wright S. (1949). The genetical structure of populations. Ann. eugenics 15, 323–354. doi: 10.1111/j.1469-1809.1949.tb02451.x 24540312

[B96] Xia X. (2018). Imputing missing distances in molecular phylogenetics. PeerJ 6, e5321. doi: 10.7717/peerj.5321 30065887 PMC6063210

[B97] Yu G. Smith D. Zhu H. Guan Y. Lam T. T.-Y. (2017). ggtree: an R package for visualization and annotation of phylogenetic trees with their covariates and other associated data. Methods Ecol. Evol. 8, 28–36. doi: 10.1111/2041-210X.12628

[B98] Zhao L. Nielsen R. Korneliussen T. S. (2022). distAngsd: fast and accurate inference of genetic distances for next-generation sequencing data. Mol. Biol. Evol. 39 (6). doi: 10.1093/molbev/msac119 PMC9234764 35647675

[B99] Zhao Z. Timofeev N. Hartley S. W. Chui D. H. Fucharoen S. Perls T. T.. (2008). Imputation of missing genotypes: an empirical evaluation of IMPUTE. BMC Genet. 9, 85. doi: 10.1186/1471-2156-9-85 19077279 PMC2636842

[B100] Zhu J. (2025). DataFusion-GDM (Melbourne, Australia: The University of Melbourne. Software). doi: 10.26188/28602953.v1

[B101] Zhu J. Giri K. Cogan N. O. Smith K. F. Jacobs J. L. (2023). Genotype-by-environment interaction analysis of dry matter yield of perennial ryegrass cultivars across south-eastern Australia using factor analytic models. Field Crops Res. 303, 109143. doi: 10.1016/j.fcr.2023.109143

[B102] Zhu J. Malmberg M. M. Shinozuka M. Retegan R. Cogan N. O. Giri K.. (2024). Ryegrass_Genotype_Allele_Frequency_Dataset (Melbourne, Australia: The University of Melbourne). doi: 10.26188/26392210.v1

