# Supplementary material for: Machine learning solutions for integrating partially overlapping genetic datasets and modelling host–endophyte effects in ryegrass (Lolium) dry matter yield estimation
Source: Front Plant Sci. 2025 May 6;16:1543956. doi: 10.3389/fpls.2025.1543956 (PMC12100933; doi:10.3389/fpls.2025.1543956)
Supplement: Supplementary File 1 — Population_Sequencing_Details. [file DataSheet1.pdf]

# Supplementary Material

**Supplementary File1**: Details of Sequencing Metrics for 80 Ryegrass Cultivars, along with Ploidy, Species, and Breeder Information

**Supplementary Table 1.** Sequencing Metrics for 80 Ryegrass Cultivars, along with Ploidy, Species, and Breeder Information

| | Sample | Ploidy | Species | Coverage | Total seqs (M) | Reads Mapped (M) | Mapped Prop (%) | Paired Prop | Breeder |
|---|---|---|---|---|---|---|---|---|---|
| 1 | 24Seven Edge | Diploid | *Lolium perenne* L. | 203.23 | 16.9 | 16.8 | 99.41% | 96.80% | DLF [c] |
| 2 | 24Seven Happe | Diploid | *Lolium perenne* L. | 202.44 | 16.7 | 16.6 | 99.40% | 97.00% | DLF [c] |
| 3 | 4Front NEA2 | Tetraploid | *Lolium perenne* L. | 87.49 | 8 | 7.9 | 98.75% | 96.20% | Barenbrug Australia |
| 4 | Alto AR37 | Diploid | *Lolium perenne* L. | 494.28 | 40.8 | 40.6 | 99.51% | 96.20% | Barenbrug Australia |
| 5 | Ansa AR1 | Diploid | *Lolium perenne* L. | 179.54 | 14.7 | 14.5 | 98.64% | 96.90% | DLF [c] |
| 6 | Ansa Happe | Diploid | *Lolium perenne* L. | 250.25 | 20.5 | 20.3 | 99.02% | 97.10% | DLF [c] |
| 7 | Arrow AR1 | Diploid | *Lolium perenne* L. | 590.28 | 49.1 | 48.7 | 99.19% | 96.50% | Barenbrug Australia |
| 8 | AusVic Nil | Diploid | *Lolium perenne* L. | 230.87 | 19.2 | 19.1 | 99.48% | 96.90% | Vicseeds |
| 9 | Avalon AR1 | Diploid | *Lolium perenne* L. | 202.26 | 16.8 | 16.7 | 99.40% | 97.30% | AGF Seeds |
| 10 | Avalon Nil | Diploid | *Lolium perenne* L. | 205.81 | 17.1 | 17 | 99.42% | 97.10% | AGF Seeds |
| 11 | Award WT | Diploid | *Lolium perenne* L. | 300.51 | 24.8 | 24.6 | 99.19% | 96.10% | Upper Murray Seeds |
| 12 | BanquetII Endo5 | Tetraploid | *Lolium* x boucheanum | 218.67 | 17.9 | 17.8 | 99.44% | 97.20% | DLF [c] |
| 13 | Barberia Nil | Diploid | *Lolium multiflorum* Lam. | 213.19 | 18 | 17.9 | 99.44% | 96.20% | Barenbrug Australia |
| 14 | Base AR1 | Tetraploid | *Lolium perenne* L. | 256.11 | 21.5 | 21.3 | 99.07% | 95.90% | DLF [c] |
| 15 | Base AR37 | Tetraploid | *Lolium perenne* L. | 134.46 | 11.1 | 11 | 99.10% | 96.80% | DLF [c] |
| 16 | Bealey NEA2 | Tetraploid | *Lolium perenne* L. | 268.84 | 22.2 | 22 | 99.10% | 96.80% | Barenbrug Australia |
| 17 | BL001 [b] | Diploid | *Lolium* ssp. | 571.58 | 47.2 | 46.9 | 99.36% | 95.90% | Breeding line |
| 18 | BL002 [b] | Diploid | *Lolium* ssp. | 736.96 | 60.8 | 60.5 | 99.51% | 96.30% | Breeding line |
| 19 | BL003 [b] | Diploid | *Lolium* ssp. | 545.74 | 45.4 | 45.1 | 99.34% | 96.40% | Breeding line |
| 20 | BL004 [b] | Diploid | *Lolium* ssp. | 718.18 | 59.8 | 59.5 | 99.50% | 96.20% | Breeding line |
| 21 | BL005 [b] | Diploid | *Lolium* ssp. | 565.65 | 46.7 | 46.5 | 99.57% | 96.60% | Breeding line |
| 22 | BL006 [b] | Diploid | *Lolium* ssp. | 621.03 | 51.7 | 51.3 | 99.23% | 96.20% | Breeding line |
| 23 | BL007 [b] | Diploid | *Lolium* ssp. | 468.99 | 38.7 | 38.5 | 99.48% | 96.30% | Breeding line |
| 24 | BL008 [b] | Diploid | *Lolium* ssp. | 697.13 | 58 | 57.6 | 99.31% | 96.60% | Breeding line |
| 25 | BL009 [b] | Diploid | *Lolium* ssp. | 513.46 | 42.4 | 42.1 | 99.29% | 95.60% | Breeding line |
| 26 | BL010 [b] | Diploid | *Lolium* ssp. | 389.35 | 32.4 | 32 | 98.77% | 96.00% | Breeding line |
| 27 | BL011 [b] | Diploid | *Lolium* ssp. | 549.73 | 45.4 | 44.9 | 98.90% | 96.00% | Breeding line |
| 28 | BL012 [b] | Tetraploid | *Lolium* ssp. | 640.79 | 53.4 | 53.2 | 99.63% | 96.30% | Breeding line |
| 29 | BL013 [b] | Diploid | *Lolium* ssp. | 460.13 | 38 | 37.7 | 99.21% | 96.40% | Breeding line |
| 30 | BL014 [b] | Tetraploid | *Lolium* ssp. | 385.51 | 32.1 | 31.9 | 99.38% | 96.20% | Breeding line |

| | | | | | | | | | | |
|---|---|---|---|---|---|---|---|---|---|---|
| 31 | BL015 [b] | Tetraploid | *Lolium* ssp. | 543.29 | 44.9 | 44.5 | 99.11% | 95.60% | Breeding line |
| 32 | BL016 [b] | NA | *Lolium perenne* L. | 546.97 | 45.2 | 44.9 | 99.34% | 96.20% | Breeding line |
| 33 | BL017 [b] | Diploid | *Lolium multiflorum* Lam. | 579.81 | 48.4 | 48 | 99.17% | 95.20% | Breeding line |
| 34 | Bolton WT | Diploid | *Lolium perenne* L. | 276.64 | 22.8 | 22.6 | 99.12% | 97.10% | AGF Seeds |
| 35 | Boomer Nil | Diploid | *Lolium perenne* L. | 213.53 | 17.6 | 17.4 | 98.86% | 97.00% | Valley Seeds |
| 36 | Camel Nil | Diploid | *Lolium perenne* L. | 308.37 | 25.7 | 25.5 | 99.22% | 97.10% | Valley Seeds |
| 37 | Endure WT | Tetraploid | *Lolium perenne* L. | 399.34 | 33.2 | 32.9 | 99.10% | 96.90% | GrasslanzTechnology |
| 38 | Excess AR1 | Diploid | *Lolium perenne* L. | 190.2 | 15.7 | 15.6 | 99.36% | 96.90% | DLF [c] |
| 39 | Excess AR37 | Diploid | *Lolium perenne* L. | 228.32 | 19 | 18.9 | 99.47% | 97.00% | DLF [c] |
| 40 | Expo AR37 | Diploid | *Lolium perenne* L. | 301.72 | 24.9 | 24.7 | 99.20% | 96.90% | DLF [c] |
| 41 | Extreme AR1 | Diploid | *Lolium perenne* L. | 159.88 | 13.3 | 13.2 | 99.25% | 96.90% | DLF [c] |
| 42 | Fitzroy WT | Diploid | *Lolium perenne* L. | 298.51 | 24.6 | 24.4 | 99.19% | 97.00% | DLF [c] |
| 43 | Governor AR1 | Diploid | *Lolium perenne* L. | 216.24 | 18 | 17.9 | 99.44% | 96.80% | Barenbrug Australia |
| 44 | Halo AR37 | Tetraploid | *Lolium perenne* L. | 253.07 | 20.9 | 20.7 | 99.04% | 96.90% | DLF [c] |
| 45 | Helix AR1 | Diploid | Festulolium hybrids | 19.2 | 1.6 | 1.6 | 100% | 96.10% | Cropmark Seeds Limited |
| 46 | Impact2 NEA2 | Diploid | *Lolium* x boucheanum | 225.34 | 18.6 | 18.4 | 98.92% | 96.60% | Barenbrug Australia |
| 47 | Jeta AR1 | Tetraploid | *Lolium* x boucheanum | 159.46 | 13.2 | 13.1 | 99.24% | 96.60% | DLF [c] |
| 48 | Jumbuck Nil | Diploid | *Lolium perenne* L. | 576.84 | 47.2 | 46.9 | 99.36% | 95.70% | Upper Murray Seeds |
| 49 | Kamo AR37 | Diploid | *Lolium perenne* L. | 499.49 | 41.2 | 40.9 | 99.27% | 96.50% | DLF [c] |
| 50 | Kidman AR1 | Diploid | *Lolium perenne* L. | 265.48 | 21.9 | 21.7 | 99.09% | 97.00% | Barenbrug Australia |
| 51 | Kingsgate WT | Diploid | *Lolium perenne* L. | 570.08 | 47.4 | 47.1 | 99.37% | 96.00% | DLF [c] |
| 52 | Kingston WT | Diploid | *Lolium perenne* L. | 242.79 | 20.7 | 20.6 | 99.52% | 97.10% | DLF [c] |
| 53 | Legion AR37 | Diploid | *Lolium perenne* L. | 462.17 | 38.2 | 37.8 | 98.95% | 95.90% | DLF [c] |
| 54 | Matrix WT | Diploid | *Lolium perenne* L. | 286.89 | 23.9 | 23.7 | 99.16% | 96.70% | Cropmark Seeds Limited |
| 55 | Maxsyn NEA12 | Diploid | *Lolium perenne* L. | 513.46 | 42.4 | 42 | 99.06% | 96.10% | Barenbrug Australia |
| 56 | Maxsyn NEA4 | Diploid | *Lolium perenne* L. | 102.19 | 8.5 | 8.5 | 100% | 96.70% | Barenbrug Australia |
| 57 | Meridian AR1 | Diploid | *Lolium perenne* L. | 158.87 | 13.1 | 13 | 99.24% | 96.80% | Barenbrug Australia |
| 58 | Munch Nil | Tetraploid | *Lolium perenne* L. | 557.19 | 46.4 | 46.1 | 99.35% | 95.70% | Upper Murray Seeds |
| 59 | Ohau AR37 | Tetraploid | *Lolium* x boucheanum | 227.18 | 18.8 | 18.6 | 98.94% | 96.60% | DLF [c] |
| 60 | One50 AR1 | Diploid | *Lolium perenne* L. | 468.43 | 39 | 38.8 | 99.49% | 95.80% | DLF [c] |
| 61 | One50 AR37 | Diploid | *Lolium perenne* L. | 247.17 | 20.4 | 20.2 | 99.02% | 97.10% | DLF [c] |
| 62 | One50 WT | Diploid | *Lolium perenne* L. | 117.78 | 9.8 | 9.7 | 98.98% | 96.60% | DLF [c] |
| 63 | Perun Nil | Tetraploid | Festulolium hybrids | 491.2 | 40.8 | 40.6 | 99.51% | 95.90% | DLF [c] |
| 64 | Platform AR37 | Diploid | *Lolium perenne* L. | 265.31 | 22.1 | 21.8 | 98.64% | 96.00% | DLF [c] |
| 65 | Platinum Nil | Diploid | *Lolium perenne* L. | 209.54 | 17.3 | 17.2 | 99.42% | 97.20% | Valley Seeds |
| 66 | Prospect AR37 | Diploid | *Lolium perenne* L. | 255.89 | 21.3 | 21.2 | 99.53% | 96.90% | DLF [c] |
| 67 | Rely AR1 | Diploid | *Lolium perenne* L. | 526.21 | 43.4 | 43.1 | 99.31% | 95.50% | DLF [c] |
| 68 | Request AR37 | Diploid | *Lolium perenne* L. | 263.42 | 21.9 | 21.8 | 99.54% | 97.00% | DLF [c] |

| | | | | | | | | | |
|---|---|---|---|---|---|---|---|---|---|
| 69 | Revolution AR1 | Diploid | *Lolium perenne* L. | 514.07 | 42.8 | 42.5 | 99.30% | 95.70% | Cropmark Seeds Limited |
| 70 | Reward Endo5 | Tetraploid | *Lolium perenne* L. | 186.57 | 15.4 | 15.3 | 99.35% | 96.70% | DLF [c] |
| 71 | Rohan NEA2 | Diploid | *Lolium perenne* L. | 599.84 | 49.9 | 49.5 | 99.20% | 96.10% | Barenbrug Australia |
| 72 | Samson WT | Diploid | *Lolium perenne* L. | 298.24 | 24.6 | 24.4 | 99.19% | 97.00% | DLF [c] |
| 73 | SFHustle AR1 | Diploid | *Lolium perenne* L. | 193.57 | 16.1 | 16 | 99.38% | 96.80% | AGF Seeds |
| 74 | Shogun NEA12 | Tetraploid | *Lolium* x boucheanum | 150.67 | 12.6 | 12.5 | 99.21% | 96.50% | Barenbrug Australia |
| 75 | Tyson NEA4 | Diploid | *Lolium perenne* L. | 387.87 | 32 | 31.8 | 99.38% | 95.90% | Barenbrug Australia |
| 76 | Ultra AR1 | Diploid | Festulolium hybrids | 451.37 | 37.3 | 37.1 | 99.46% | 95.80% | Cropmark Seeds Limited |
| 77 | Victoca Nil | Diploid | *Lolium perenne* L. | 321.54 | 26.5 | 26.3 | 99.25% | 97.20% | Tasglobal Seeds Pty Ltd. |
| 78 | Victorian [a] WT | Diploid | *Lolium perenne* L. | 276.73 | 22.8 | 22.6 | 99.12% | 97.10% | NA [a] |
| 79 | Viscount NEA2 | Tetraploid | *Lolium perenne* L. | 265.23 | 21.9 | 21.7 | 99.09% | 97.10% | Barenbrug Australia |
| 80 | WintasII | Diploid | *Lolium multiflorum* Lam. | 288.83 | 24.1 | 24 | 99.59% | 96.40% | Tasglobal Seeds Pty Ltd. |
| | **Average** | | | **350.81** | **29.08** | **28.87** | **99.27%** | **96.50%** | |

[a] Victorian is a perennial ryegrass native to south-east Australia.

[b] BLxxx are breeding lines, which are commercially confidential and not publicly available. The authors of this paper are not authorized to share the information.

[c] DLF: DLF Seeds Australia/ PGG Wrightson Seeds (Australia) Pty Limited/ Grasslands Innovation Limited.

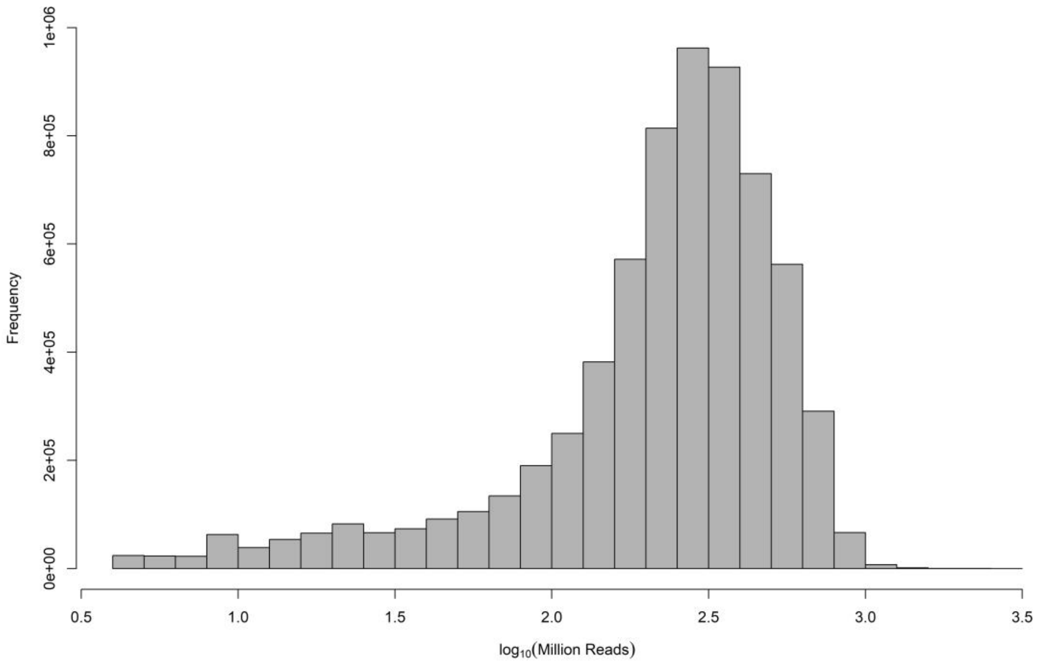


**Supplementary Figure 1.** Histogram of the number of reads at each locus after normalisation across all sequenced ryegrass samples. The x-axis represents the log10 of million reads, and the y-axis represents the frequency of occurrences. The distribution peaks around a log10 of 2.5, indicating around 316 million reads.
