# Supplementary material for: Machine learning solutions for integrating partially overlapping genetic datasets and modelling host–endophyte effects in ryegrass (Lolium) dry matter yield estimation
Source: Front Plant Sci. 2025 May 6;16:1543956. doi: 10.3389/fpls.2025.1543956 (PMC12100933; doi:10.3389/fpls.2025.1543956)
Supplement: Supplementary File 2 — Population_Genotyping_Validation. [file DataSheet2.pdf]

Supplementary Material

Supplementary_File2: Population Sequencing and Genotyping Validation

One batch of bulk DNA extraction was performed using 50 seeds of Rohan (Rohan seed bulk sequencing). Another batch of bulk DNA extraction was processed on Kidman (Kidman seed bulk sequencing) using the same procedure as Rohan. The seeds of Kidman were also germinated in seedling trays in the glasshouse with leaf samples used for plant bulk or plant individual DNA extractions: 1) one batch of bulk DNA extraction (Kidman plant bulk sequencing) was performed by cutting a 0.5 cm piece of the second new leaf at the three-leaf stage from 96 Kidman leaf samples; 2) 96 batches of individual DNA extractions were performed with each individual plant treated as a sequencing sample.

Target capture was performed on pooled libraries of each of Kidman plant bulk sequencing, Kidman seed bulk sequencing, and Rohan seed bulk sequencing, respectively. The Kidman plant individual sequencing samples were pooled into two pools, each containing 48 samples before undergoing target capture. To ensure sufficient material for sequencing, the DNA extracted from each bulk sample underwent library preparations and was pooled before target capture.

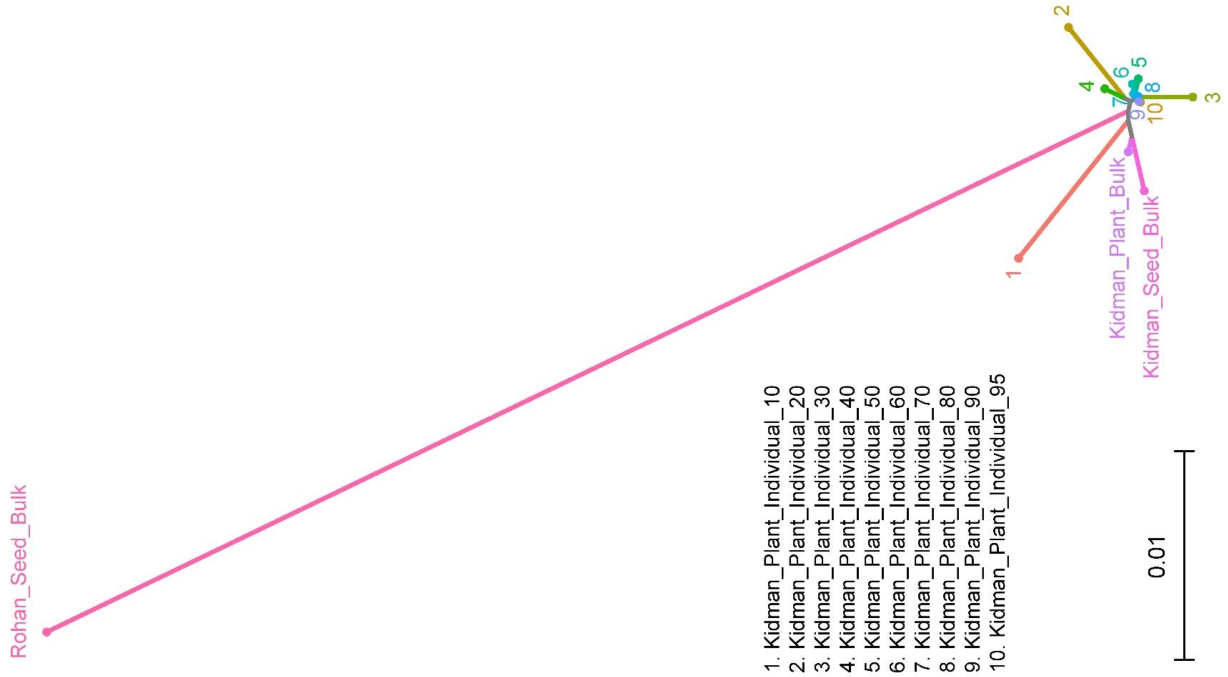

Supplementary Figure 2. An unrooted tree illustrating genetic differentiation between seed versus leaf sequencing, and individual versus bulk sequencing for Rohan and Kidman samples. Labels 1 to 10 represent genotyping analysis from pooled sequencing of 10, 20, 30, 40, 50, 60, 70, 80, 90, and 95 individuals, respectively, each derived from individual sequencing. The branches labelled Kidman_Plant_Bulk and Kidman_Seed_Bulk represent bulk sequencing samples of Kidman plants and seeds, respectively; the branch labelled Rohan_Seed_Bulk represents bulk sequencing of Rohan seeds. The scale bar indicates Nei's genetic distance of 0.01.
