# Supplementary material for: Machine learning solutions for integrating partially overlapping genetic datasets and modelling host–endophyte effects in ryegrass (Lolium) dry matter yield estimation
Source: Front Plant Sci. 2025 May 6;16:1543956. doi: 10.3389/fpls.2025.1543956 (PMC12100933; doi:10.3389/fpls.2025.1543956)
Supplement: Supplementary File 3 — Integration_of_Partially_Overlapped_GDMs. [file DataSheet3.zip › Imputation_Models.pdf]

Supplementary Material

Supplementary_File3: Integration of Partially Overlapped Genetic Distance Matrices - The Explored Imputation Models for Structural Missingness

1 Regression Trees (RT):

Regression Trees was used for imputation due to its ability to handle non-linear relationships and potential interactions between populations; plus, it constructed a decision tree by recursively splitting the dataset based on features that minimize the variance at each node. These features made it suitable for continuous values such as Nei's genetic distances. During imputation, the tree was constructed using the valid data (i.e., the observed Nei's genetic distances or the propagated predicted Nei's genetic distances) by minimizing the variance in node t:

$$V(t) = \frac{1}{N_t}\sum_{i \in t}(\boldsymbol{y}_i - \bar{y}_t)^2$$

where, $\boldsymbol{y}_i$ are the valid Nei's genetic distances in a given leaf node t; $\bar{y}_t$ is the mean of $\boldsymbol{y}_i$; N_t is the number of $\boldsymbol{y}_i$ in the node t.

The imputed value for a missing data (NA) was calculated by $\bar{y}_t$ in the corresponding leaf node (t) of the tree:

$$y(t) = \bar{y}_t = \frac{1}{N_t}\sum_{i \in t}\boldsymbol{y}_i$$

2 Random Forest (RF):

Random Forest is an ensemble method that builds multiple decision trees and merges them to get a more accurate and stable prediction. Each tree in the forest was built using a random subset of the data, and the final imputed value was the average prediction from all trees. It was effective for working with Nei's genetic distances due to its ability to capture complex interactions among populations and reduce the degree of overfitting. The imputed distance was the average prediction from all trees in the forest, given as:

$\hat{y} = \frac{1}{n}\sum_{i=1}^{n} \boldsymbol{y}_i$

where, $\hat{y}$ is the imputed Nei's genetic distance; n is the number of trees in the forest; $\boldsymbol{y}_i$ is the prediction from the i-th tree.

3 Lasso Regression (Lasso):

Lasso Regression was used for imputing Nei's genetic distances due to its ability to handle multi-dimensional data and exclude certain dimensions (i.e., the populations) by shrinking their coefficients to zero. This was done by minimizing the cost function with an L1 regularization term:

$cost\_function = \sum_i \left(\boldsymbol{y}_i - \beta_0 - \sum_j \boldsymbol{\beta}_j \boldsymbol{x}_{ij}\right)^2 + \lambda \sum_j \left|\boldsymbol{\beta}_j\right|$

where, y_i are the valid Nei's genetic distances; β_0 is the intercept; $\boldsymbol{\beta}_j$ are the regression coefficients; $\boldsymbol{x}_{ij}$ are the predictor populations; λ is a regularisation parameter; $\left|\boldsymbol{\beta}_j\right|$ is the absolute value of the j-th coefficient.

4 K-Nearest Neighbors (KNN):

K-Nearest Neighbors imputed missing Nei's genetic distances based on the k nearest neighbours in a Euclidean space. This method assumes genetic distances of similar populations are clustered together. The imputed Nei's genetic distances ($\hat{y}$) were given by the average of the valid Nei's genetic distances ($\boldsymbol{y}_i$) of the k nearest neighbors:

$\hat{y} = \frac{1}{k}\sum_{i=1}^{k} \boldsymbol{y}_i$

The Euclidean distance $\Delta\left(\boldsymbol{x}_i, \boldsymbol{x}_j\right) = \sqrt{\sum_{v=1}^{(p+q)} \left(\boldsymbol{x}_{iv} - \boldsymbol{x}_{jv}\right)^2}$

where, $(p + q)$ is the sum of the number of observed populations and propagated predicted populations; v is the index of populations.

5 Predictive Mean Matching (PMM):

Predictive Mean Matching was performed by identifying valid Nei's genetic distance values whose predicted means are closest to the predicted mean of the missing values (NAs), and randomly selecting one as the imputed value:

$$\boldsymbol{y}_{ij} = \beta_0 + \sum_{k=1}^{p+q-1} \boldsymbol{\beta}_k x_{ik} + \boldsymbol{\varepsilon}_i$$

where, $\boldsymbol{y}_{ij}$ is the j-th population to be predicted paired with the i-th population; β_0 is the intercept; $\boldsymbol{\beta}_k$ are coefficients for the predictors $\boldsymbol{x}_{ik}$; p and q are the indices of the observed populations and propagated predicted populations, respectively; $\boldsymbol{\varepsilon}_i$ is the error term for the i-th populatio.

For each missing case y_{mis}, identify donors $Set(\boldsymbol{y}_{obs})$, whose predicted values $\hat{y}_{obs}$ are close to the $\hat{y}_{mis}$; impute the missing cases y_{mis} by randomly selecting a donor from $Set(\boldsymbol{y}_{obs})$:

$$\hat{y}_{mis} = y_{obs}, \text{ where } y_{obs} \in Set(\boldsymbol{y}_{obs}) = \left\{ \boldsymbol{y}_{obs} \middle| \underset{i,j \in obs}{arg\ min}(|\hat{y}_{mis} - \hat{y}_{obs}|) \right\}$$

6 Weighted Predictive Mean Matching (WPMM):

Weighted Predictive Mean Matching is a variant of PMM that assigns different weights ($\boldsymbol{\omega}_k$) to donors ($Set(\boldsymbol{y}_{obs})$) based on the closeness of $\hat{y}_{obs}$ to $\hat{y}_{mis}$, providing a more refined imputation for Nei's genetic distances. The weights were given as: $\omega_k = exp\left(-\frac{|\hat{y}_{mis}-\hat{y}_{obs}|^2}{2}\right)$ and were normalized by $\omega_k = \frac{\omega_k}{\sum_k \omega_k}$. Then the missing cases y_{mis} was imputed by taking the weighted average of the donors: $\hat{y}_{mis} = \sum_k \omega_k\, y_{obs}$.

7 Random Sampling (SAMPLE):

Random Sampling randomly selected a valid Nei's genetic distance to replace the missing values. The NAs were imputed by $\hat{y}_{ij} = y_{ij}$, where the probability of selecting a certain y_{ij} is $P(\boldsymbol{y}_{ij}) = \frac{1}{p+q-2}$, $i \neq j$. This method is simple but may not capture the underlying genetic structure.

8 Mean (Baseline):

As a baseline, mean imputation replaced missing Nei's genetic distances with the mean of the observed values (not including propagated predicted values) per row.

$$\hat{y}_{ij} = \frac{1}{p}\left(\sum y_j\right)_i$$
