# Supplementary material for: Machine learning solutions for integrating partially overlapping genetic datasets and modelling host–endophyte effects in ryegrass (Lolium) dry matter yield estimation
Source: Front Plant Sci. 2025 May 6;16:1543956. doi: 10.3389/fpls.2025.1543956 (PMC12100933; doi:10.3389/fpls.2025.1543956)
Supplement: Supplementary File 4 — Information_of_the_2_Genetic_Datasets. [file DataSheet4.pdf]

Supplementary Material

Supplementary_File4: Metadata of Ryegrass72_2024 and Ryegrass63_2016.

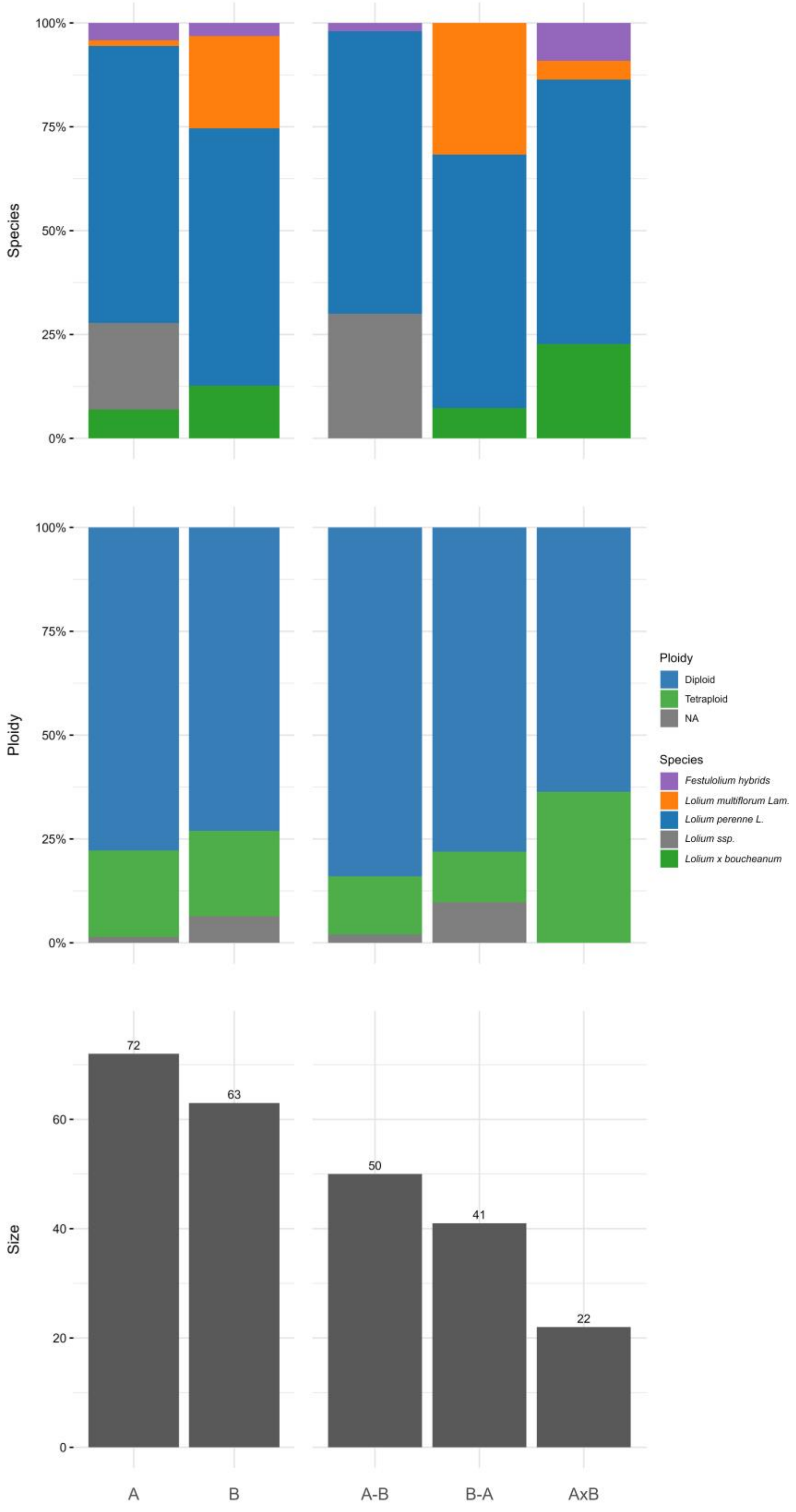

Supplementary Figure 3. Metadata visualization of Ryegrass72_2024 (***A***) and Ryegrass63_2016 (***B***) datasets, including species composition, ploidy levels, and set/intersection sizes. ***A***: 72, ***B***: 63, ***A-B***: 50 (unique to ***A***), ***B-A***: 41 (unique to **B**), ***A×B***: 22 (shared by ***A*** and ***B***). Species and ploidy compositions are shown for each set or intersection.

The datasets Ryegrass72_2024 (***A***) and Ryegrass63_2016 (***B***) comprise 72 and 63 ryegrass populations, respectively, and share 22 common populations, as shown in the intersection plot (Supplementary Figure 3). Species and ploidy compositions varied between the datasets. Both datasets are dominated by *Lolium perenne* L., while *B* also shows a prevalence of *Lolium multiflorum* Lam. The intersection (***A***×***B***) contains all the spp. of the two datasets and are primarily composed of *Lolium perenne* L. Regarding ploidy, ***A***×***B*** maintains a similar ploidy composition to both datasets, with diploids being more common than tetraploids.
