# Supplementary material for: Machine learning solutions for integrating partially overlapping genetic datasets and modelling host–endophyte effects in ryegrass (Lolium) dry matter yield estimation
Source: Front Plant Sci. 2025 May 6;16:1543956. doi: 10.3389/fpls.2025.1543956 (PMC12100933; doi:10.3389/fpls.2025.1543956)
Supplement: Supplementary File 5 — M. [file DataSheet5.zip › Supplementary_File5.1_M_Visualization.pdf]

Supplementary Material

Supplementary_File5: The merged and the imputed Nei’s genetic distance matrices.

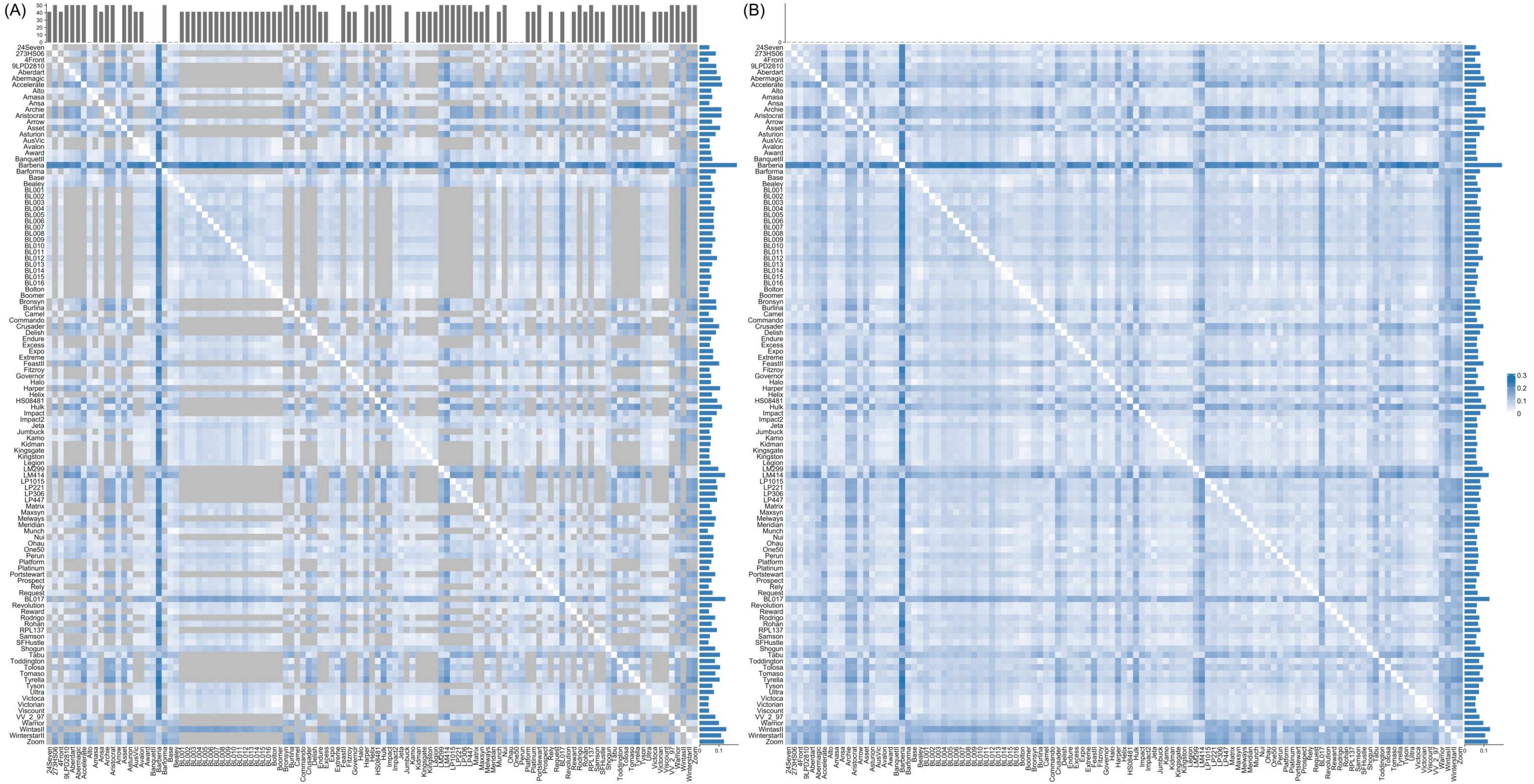

Supplementary Figure 4. (A) The symmetric heatmap of the merged Nei's genetic distance matrices of Ryegrass72_2024 (A) and calibrated Ryegrass63_2016 (B'). The bar plot at the top displays the proportion of NAs in the genetic configuration of each population. The bar plot on the right shows the mean of genetic distances for each population, providing a general overview of the genetic dissimilarity. **(B)** The symmetric heatmap of the imputed Nei's genetic distance matrix $\boldsymbol{M}^*$. Colour intensity indicates the pairwise genetic distances.
